# Why we should care about moral foundations when preparing for the next pandemic: Insights from Canada, the UK and the US

**DOI:** 10.1371/journal.pone.0285549

**Published:** 2023-05-12

**Authors:** Lizette Pizza, Samuel Ronfard, John D. Coley, Deborah Kelemen

**Affiliations:** 1 Department of Psychological and Brain Sciences, Boston University, Boston, Massachusetts, United States of America; 2 Department of Psychology, University of Toronto at Mississauga, Mississauga, Ontario, Canada; 3 Department of Psychology, Northeastern University, Boston, Massachusetts, United States of America; 4 Department of Marine & Environmental Sciences, Northeastern University, Boston, Massachusetts, United States of America; Queen Mary University of London, UNITED KINGDOM

## Abstract

Health behaviors that do not effectively prevent disease can negatively impact psychological wellbeing and potentially drain motivations to engage in more effective behavior, potentially creating higher health risk. Despite this, studies linking “moral foundations” (i.e., concerns about harm, fairness, purity, authority, ingroup, and/or liberty) to health behaviors have generally been limited to a narrow range of behaviors, specifically effective ones. We therefore explored the degree to which moral foundations predicted a wider range of not only effective but ineffective (overreactive) preventative behaviors during the COVID-19 pandemic. In Study 1, participants from Canada, the United Kingdom, and the United States reported their engagement in these preventative behaviors and completed a COVID-specific adaptation of the Moral Foundations Questionnaire during the pandemic peak. While differences occurred across countries, authority considerations consistently predicted increased engagement in both effective preventative behaviors but also ineffective overreactions, even when controlling for political ideology. By contrast, purity and liberty considerations reduced intentions to engage in effective behaviors like vaccination but had no effect on ineffective behaviors. Study 2 revealed that the influence of moral foundations on U.S participants’ behavior remained stable 5-months later, after the pandemic peak. These findings demonstrate that the impact of moral foundations on preventative behaviors is similar across a range of western democracies, and that recommendations by authorities can have unexpected consequences in terms of promoting ineffective—and potentially damaging—overreactive behaviors. The findings underscore the importance of moral concerns for the design of health interventions that selectively promote effective preventative behavior.

## Introduction

As a result of continuous globalization, national borders have become more diffuse, and infectious diseases have spread more rapidly across countries [1, 2]. In the last two decades, the world has seen multiple disease outbreaks emerging in different locations (e.g., H1N1 pandemic, Ebola, COVID-19), provoking profound health and social-economic crises along with widespread fear. Thus, it is critical that governments develop and implement effective strategies to control and prevent the emergence of infectious diseases [3]. This is difficult given that many factors influence how resources are allocated to support public health. Psychological factors that promote effective public health behaviors [4–6] and reduce deleterious or ineffective behavioral change are especially important.

Given its magnitude, the COVID-19 pandemic created an opportunity for understanding the impact of various psychological factors related to preventative health behavior change. Although variables like trust in science, misinformation, and scientific knowledge about the virus have been shown to predict COVID-related health behavior [7, 8], we argue here that special attention should be directed to the role of moral concerns. Moral concerns implicitly impact people’s judgments, play an important role in their health decision-making [9–11], and robustly predict behavior [12]. In fact, political ideology—which is often treated as a proxy for moral values—is sometimes more predictive of intentions to modify health behavior than scientific knowledge [13].

Although several researchers have found evidence of the important role of moral values in predicting participants’ engagement in preventative behaviors, to our knowledge no research has assessed whether moral concerns also influence participants’ engagement in less desirable behaviors, that is, behaviors that are ineffective like wearing a mask when alone. Specifically, although a study associated with this one found that U.S. participants with greater biological knowledge were less likely to engage in ineffective behaviors [14], it remains unknown whether moral values also influence people’s tendency to engage in ineffective behaviors. This is important because effective public health interventions need to reduce behaviors that are irrelevant to illness transmission but may have other deleterious effects (e.g., heightened anxiety, increased social isolation) while promoting behaviors that actually reduce illness transmission. In this research, we draw on Moral Foundations Theory [15], to explore the influence of people’s abstract moral considerations (i.e., “moral foundations”)—concepts such as personal and interpersonal harm, ingroup loyalty, and authority—on their self-reported health effective and ineffective behaviors when reasoning about various pandemic-related contexts and at different pandemic timepoints.

In Study 1, we examined whether specific moral foundations predicted people’s engagement in preventative behaviors and their intentions to vaccinate in the US and two other western countries (Canada and the UK) during the initial peak of the COVID-19 pandemic of December 2020 and January 2021 when daily mortality rates were high and COVID vaccinations were not yet available. In Study 2, we explored the stability of our earlier results at a later time point in the US, specifically, once the pandemic was past its initial deadliest peak and after vaccine programs started being rolled out for adults.

### Moral foundations and health behaviors

According to Haidt and Joseph [15], moral foundations are innate, universal, affectively-based psychological systems that constitute the building blocks of morality with cultures varying in the weight accorded to each of them [16–18]. Five moral foundations have been identified: The *harm* foundation focuses on concern about others’ suffering as well as personal welfare, and the *fairness* foundation focuses on concerns about equal resource distribution, although these two foundations are usually clustered together as individualizing moral foundations. The remaining three foundations involve concerns that bind social groups (i.e., binding foundations). The *authority* foundation concerns the importance of showing obedience to rules and to others within a social hierarchy; the *ingroup* foundation relates to the virtues of loyalty and willingness to sacrifice for the group; and the *purity* foundation includes concerns about preserving naturalness or spiritual and physical cleanliness. Because—via the emotion of disgust—the purity foundation promotes avoidance of contamination by potentially invisible harms, it has been proposed as an evolutionary adaptation for avoiding disease transmission [18]. It is therefore particularly relevant in a pandemic situation. A sixth moral foundation—*liberty*—has also been recently proposed. It captures concerns related to an individual’s right to self-determine (i.e., be left alone) and resist oppression. Liberty is unique in that it can act as a sole guiding value at the expense of other moral foundations [19]. Given its significance, plus its relatively understudied influence on decision-making, it was a focus in this research.

Prior research on COVID-19 has found that, in general, people who value foundations such as care for others or fairness tend to comply with effective, recommended, COVID-19 preventative behaviors [10, 20–22]. This is presumably because those individuals prioritize staying healthy to protect those whose health is more vulnerable and tend to trust scientific experts’ recommendations. In contrast, in the US, non-compliance is associated with preferences for liberty over equality presumably because some individuals weigh their right to personal freedom over their sense of duty to others’ health leading them to downplay the severity of the pandemic [9]. Additional research—mainly conducted in the US—on people’s reasoning about public health measures like vaccination has also revealed associations with moral foundations. The individualizing foundations, as well as ingroup loyalty, have been found to be related to higher pro-vaccination attitudes [23, 24], while vaccine hesitancy has been related to liberty concerns—insofar as government vaccine mandates are seen to violate personal rights and civil liberties—and especially to purity considerations presumably due to individuals’ assumption that vaccines are artificial and that their components might contaminate the body and disturb the pure process of naturally building up immunity [25].

Most of the previous research has measured people’s personal weighing of different moral foundations using the Moral Foundations Questionnaire (MFQ) [26]. The MFQ asks participants to rate the relevance of foundation-related concerns to their general decision-making. However, given that the pandemic was an unexpected and novel situation, the MFQ might be too general and, therefore, unable to identify specific contextual patterns of the situation. For instance, the MFQ does not differentiate between concerns about harming others versus harming the self—a distinction that is highly relevant during a pandemic because these variables might play different roles in individuals’ preventative behaviors. Consequently, we created a modified version of the MFQ that focused specifically on the pandemic context for this study.

Furthermore, prior research on preventative behaviors and moral foundations has tended to cluster together concerns like ingroup, authority and purity [23] because they are binding foundations that often act similarly in context of other topics (e.g., incest). However, in context of a pandemic-relevant preventative behavior like vaccination, these binding foundations (e.g., authority and purity) might operate in profoundly different and potentially opposite ways. To understand these subtleties of prediction in the health context in the present study, we therefore analyzed the predictive role of each foundation independently.

Finally, and importantly, prior research demonstrating that moral concerns play an important role in predicting infection prevention behaviors has not explored their role in promoting ineffective behaviors, that is, actions that may be consistent with specific moral foundations (e.g., wearing a mask when completely alone; compulsive clothes washing, extreme social isolation) but that public health research suggests are not helpful in transmission prevention and which potentially represent dysfunctional hyper-reactive overgeneralizations of public health guidance. Insofar as they reflect and promote anxiety, these ineffective overreactions have potentially deleterious effects on long-term mental health [27]. While our prior research has shown that lower levels of biological knowledge may predict such behaviors [14] it is an open question how moral considerations influence them.

### Moral foundations and cultural considerations

The ways in which moral concerns influence behavior might not always generalize even across relatively similar cultures, since human moral systems result from specific social and cultural experiences as well as adaptive pressures [16]. Before the pandemic, most studies exploring relationships between health behaviors and moral foundations tended to be conducted in the US (but see Rossen et al. [28]). This is problematic because the US is singular even among western nations. Relative to other western societies, the US tends to have higher levels of individual religiosity, and places stronger emphasis on individual freedom [29, 30]. U.S. findings therefore might not generalize to other countries. Fortuitously, the global context of the COVID-19 pandemic created an opportunity to explore whether different countries faced with similar acute challenges showed parallel patterns of response to them.

Prior research has been equivocal over whether patterns found in the US during the pandemic have generalizability beyond its boundaries [31, 32] (but see Schmidtke et al. [33]). One reason for this lack of consistency is the distinctive and marked pattern of polarization that occurred in the US relative to other even closely related western countries (e.g., the UK): For example, in the US, conservatives reported resistance to a whole range of behaviors including mask-wearing, hand washing, and willingness to vaccinate [13, 34]. They tended to minimize the threat posed by the pandemic and in turn were more likely to contract COVID-19 [35]. By contrast, in a non-US international sample, conservatives were generally *more* likely to endorse protective behavior like mask-wearing than liberals [36] and so the same COVID-19 transmission pattern did not hold. Given the strong association between political ideology and moral foundations in the US—with conservatives usually weighing the binding foundations of ingroup loyalty, authority, and purity as more relevant than liberals [17]—it is possible that correlations between political ideology and health behaviors in US reflect a culturally distinctive pattern in underlying moral reasoning.

Although Canada and the UK are Western nations connected to the US via shared language, history, and traditions, they differ from the US in ways that might impact the kind of abstract foundations weighed in reasoning about illness, health, and the pandemic. In addition to having a reduced narrative emphasis on individual rights and liberties [30], both differ from the US by having long-established government-funded universal healthcare systems rather than privately-funded healthcare. Furthermore, although both the US and UK–unlike Canada–had politically conservative government leadership during the pandemic, the rhetoric around the pandemic in both the UK and Canada was far less politicized and overtly polarized than in the US [13, 37]. For example, in the UK and Canada, political leaders strongly promoted compliance with scientifically recommended preventative behaviors and acknowledged the serious health threat posed by COVID-19. Canada and UK therefore represented highly relevant contexts in which to examine the generalizability of moral foundation effects on health behaviors during an international emergency.

### The present research

The current studies examine how moral foundations relate to self-reported effective and ineffective preventative behaviors. In our preregistration of this research, we hypothesized that moral foundations would relate to effective preventative behaviors in each of three western countries (Canada, the UK, and the US), but it was an open question which foundations would be most predictive in each cultural context. By contrast, when considering ineffective preventative behaviors—overreactive behaviors that have not been studied to this point—and vaccine intentions, we anticipated a special role for the purity foundation given its relationship to disgust and pathogen avoidance [38]. Specifically, we hypothesized that purity beliefs might predict increases in ineffective behavior due to heightened fears about infection but decreases in intentions to vaccinate due to concerns about the tainting effects of vaccination ingredients. To test these hypotheses, in Study 1, we conducted an online study in the US, UK, and Canada at the peak of the pandemic, using an adapted contextualized version of the MFQ and questions about participants’ engagement in effective and ineffective preventative behaviors.

Because vaccination against COVID-19 was not publicly available during Study 1, our analyses separated participants’ reports of actual preventative behavior (e.g., handwashing) from self-reports of their notional preventative behavior (i.e., their intentions to vaccinate). Furthermore, given that moral foundations are argued to represent culturally-adapted abstract moral principles, we hypothesized that the relationships found in Study 1 would be relatively stable across time. To test this hypothesis, we conducted Study 2 in the US after the pandemic peak using materials adapted from Study 1. The only change we expected concerned the influence of the authority foundation on preventative behaviors given the relaxing of some official mandates as COVID-19 deaths dropped.

The specific analyses conducted for this paper were preregistered at: https://osf.io/ucn2m/?view_only=42fd4324a0f3421885c13b4519c7ec90. The surveys for Study 1 and Study 2 were part of a larger online study of adults’, specifically, parents’ reasoning about COVID-19 that is preregistered at: https://osf.io/wqvyr?view_only=6390c34605b6498fa7db91a5605d6da2 (Study 1); https://osf.io/sedx5?view_only=1f07463e8fab45a5836062f0bb935c39 (Study 2).

## Study 1

### Method

#### Participants

Participants were recruited through the online platforms Prolific and Centiment in Canada (N = 176), the UK (N = 139), and the US (N = 138). Sample size was the same as for the overall project of which this study was a part. For the project, it was estimated from power analyses that a sample size of 131 participants in each country would achieve 80% power in the analyses. To be included, participants needed to be residents of each country, and not have participated in previous related studies with our labs. Because this study was part of a broader project on reasoning about COVID-19 among parents, all participants had elementary-school-aged children, and we included questions to tap into moral foundations guiding adults’ preventative reasoning concerning children. For participants recruited through the Prolific recruitment platform, the larger Qualtrics survey included five attention checks, and any participant who failed more than one was excluded from the final sample. However, Canadian participants (44%) recruited through the Centiment platform had two attention checks and were excluded when failing one of them. This difference occurred because we needed to use more than one platform to recruit a big enough sample of parents in Canada but each platform had different attention check and retention criteria. Additionally, we excluded participants who failed at least one of four additional checks in the COVID-19 Moral Foundations Questionnaire (adapted from original MFQ attention checks). In total, 44% of participants in Canada, 8% in the UK, and 15% in the US were excluded for failed attention checks. Although the criteria set by the recruitment platform in Canada resulted in a higher exclusion rate, the Canadian sample did not significantly differ from our other samples in key demographics (see Table 1). Participants leaned liberal politically on a 1–5 composite score scale (5 = high socio-economic conservatism: US *M* = 2.6, *SD* = 1.1; Canada *M* = 2.7 *SD* = 1.0; UK *M* = 2.6 *SD* = 0.8), and generally had at least a college degree (69% in the US, 80% in Canada, and 63% in the UK). All participants provided informed written consent and the survey was approved by Boston University institutional IRB (Approval number: 2350E).

**Table 1:** Demographics of each sample in Studies 1 and 2.

Country	N	%Women	Age (years)	Self-reported Race
**Study 1**				
Canada	176	57%	M = 38 SD = 6.19	Black 5%, East Asian 11%, Latino 2%, Mixed 5%, South Asian 9%, White 65%
UK	139	65%	M = 37, SD = 5.98	Black 1%, Mixed 2%, South Asian 6%, White 91%
US	138	58%	M = 36, SD = 5.84	Black 7%, East Asian 3%, Latino 5%, Mixed 9%, South Asian 4%, White 73%
**Study 2**				
US	170	64%	M = 36, SD = 6.11	Black 7%, East Asian 2%, Latino 1%, Mixed race 5%, South Asian 1%, White 82%

Data collection started in late December 2020 just as the number of COVID-19 cases (approximately 4 million) and deaths (over 69,000) were at a peak globally since the onset of the pandemic in Fall 2019 [39]. At the time, it was public knowledge that several COVID vaccines had been developed, with the WHO authorizing emergency vaccine use for select adults, although child vaccination was not authorized yet [40, 41].

Finally, in addition to focusing on three related Western countries, for this study, we also collected data via Centiment in India to include a non-western country. However, once our pre-registered exclusion criteria were applied, an unexpectedly large number of participants had to be excluded, leaving only a small sample (N = 48) from which it is hard to generalize. The results from this smaller sample are therefore not presented in the paper and instead are included as exploratory data in Tables K to M of the S1 File (see supporting information section).

#### Materials and procedures

The present study presented three sections of a larger Qualtrics survey in fixed order. These focused on (1) participants’ self-reported engagement in effective and ineffective COVID-19 preventative behaviors, (2) their intentions to vaccinate, and (3) their answers to a COVID-19 moral foundations questionnaire. The full survey took about 25 minutes to complete.

#### Engagement in effective and ineffective preventative behaviors

Effective (helpful) and ineffective (non-helpful, overreactive) preventative behaviors were designated based on scientific information about the COVID transmission mechanism established by December 2020. Specifically, it had been confirmed by then that the coronavirus was transmitted via droplet infection, with indoor mask use and social distancing known as the best strategies to decrease viral spread [42–44]. In contrast, wearing a face mask in outdoor spaces when alone or capable of maintaining more than 6 ft. distance from others was not considered helpful.

Participants’ engagement in effective and ineffective preventative behaviors was assessed with yes/no questions about mask-wearing in seven situations. In four of the situations, mask-wearing was effective (e.g., “when grocery shopping”) while in three others it was ineffective (e.g., “when I am outdoors alone”).

Next, participants answered nine rating scale questions about social-distancing behaviors which were reverse coded. Five of the behaviors were effective (e.g., “(avoid) shaking other people’s hands”), and four of them were ineffective (e.g., “(avoid) borrowing tools/objects from my neighbor”). Participants rated the frequency of engagement with those behaviors relative to pre-pandemic times from 1 (*much less frequently*) to 5 (*much more frequently*).

Finally, we asked participants eight questions about washing behaviors, half of them were effective (e.g., “Wash my hands for 20 seconds”) and the rest were ineffective (e.g., “Wash my children’s clothes”) and they rated the frequency of engagement in them relative to pre-pandemic levels, on the same scale as the social-distancing behaviors.

Items in each section were presented in random order. From these sets of questions, two composite scores were generated for effective and ineffective preventative behaviors respectively. The composites were obtained after standardizing each item and obtaining the item mean for each behavior. Before creating the composites, four items (one ineffective and three effective) were removed because they had been difficult for participants to interpret. After exclusions, we had 10 effective preventative behavior items and 10 ineffective preventative behavior items. See Table A in S1 File for a list of all items, including exclusions.

#### Vaccine intentions

Participants were asked two questions about their intentions to vaccinate, concerning themselves and their child. In both cases, they answered a forced-choice question (e.g., “Now that vaccines are becoming publicly available, are you intending to take the vaccine?”).

#### COVID-19 moral foundations questionnaire

Participants were presented with four hypothetical decision-making scenarios about COVID-19 behaviors. The scenarios concerned mask-wearing, social distancing, intentions to personally vaccinate, and intentions to vaccinate their child (see a sample scenario in Table 2). Participants rated the degree to which they would weigh each of the 6 moral foundations as relevant in each of the 4 scenarios from 1 (*not at all relevant*) to 6 (*extremely relevant*). The moral foundations probed were: harm to others, harm to self (personal risk), authority, ingroup loyalty, purity, and liberty. The scenarios were presented in a fixed order, but the rating items were presented in random order, and we calculated an average score for each moral foundation across all four scenarios. Importantly, although moral foundation phrasings were based on the original MFQ [26], our measure departed from the MFQ in that participants’ rating of the personal relevance of each moral value was not abstracted and general (e.g., “people should not do things that are disgusting, even if no one is harmed”) but contextualized to each COVID-19 scenario being considered (see Table 2; specific wording of all scenarios and items is available in Table B in S1 File). Contextualizing the MFQ allowed us to identify which moral foundations have the most relevance for preventative behaviors during a novel worldwide health emergency that triggered very different governmental reactions and levels of public polarization in different countries.

**Table 2:** Sample COVID-19 MFQ mask-wearing scenario and its rating response items.

Mask wearing scenario	
One of your friends invited you to a party with a bunch of people. When you decide whether or not to wear a mask, to what extent are the following considerations likely to be relevant to your thinking?” (1 = *not at all relevant* to 6 = *extremely relevant*)	
Foundation	Item
Harm to others	Whether someone (apart from myself) is likely to get severely sick.
Harm to self	Whether I am likely to get sick.
Authority	Whether I am respecting the rules established by my government.
Ingroup	Whether I am betraying the values of my community or group of friends.
Purity	Whether other people going to the party are likely to be pure and decent.
Liberty	Whether I feel I am exercising my liberty.

Other departures from the original MFQ were the inclusion of ratings for moral concerns about liberty, because we expected them to be relevant [19], but the exclusion of fairness concerns because we could not easily adapt the MFQ’s original fairness item to each of our four COVID-19 scenarios. Also, we included ratings for concerns about harm to self so we could distinguish them from concerns about harming others within the COVID-19 context. The distinction is important to investigate given that previous research has suggested that framing health campaigns in terms of harm to the self versus harm to others can lead to differential effectiveness [45, 46].

### Results

Cronbach’s alpha confirmed the reliability of the scores for effective and ineffective preventative behaviors (ranging from .73 to .89) and moral foundation scores (ranging from .78 to .92), which showed acceptable to excellent levels of internal consistency in each country. An exception to this was for the purity foundation composite. However, excluding one item resulted in acceptable reliability levels in each country (ranging from .67 to .74), so we retained that composite given our preregistration.

#### Moral foundations across situations

**Fig 1:**
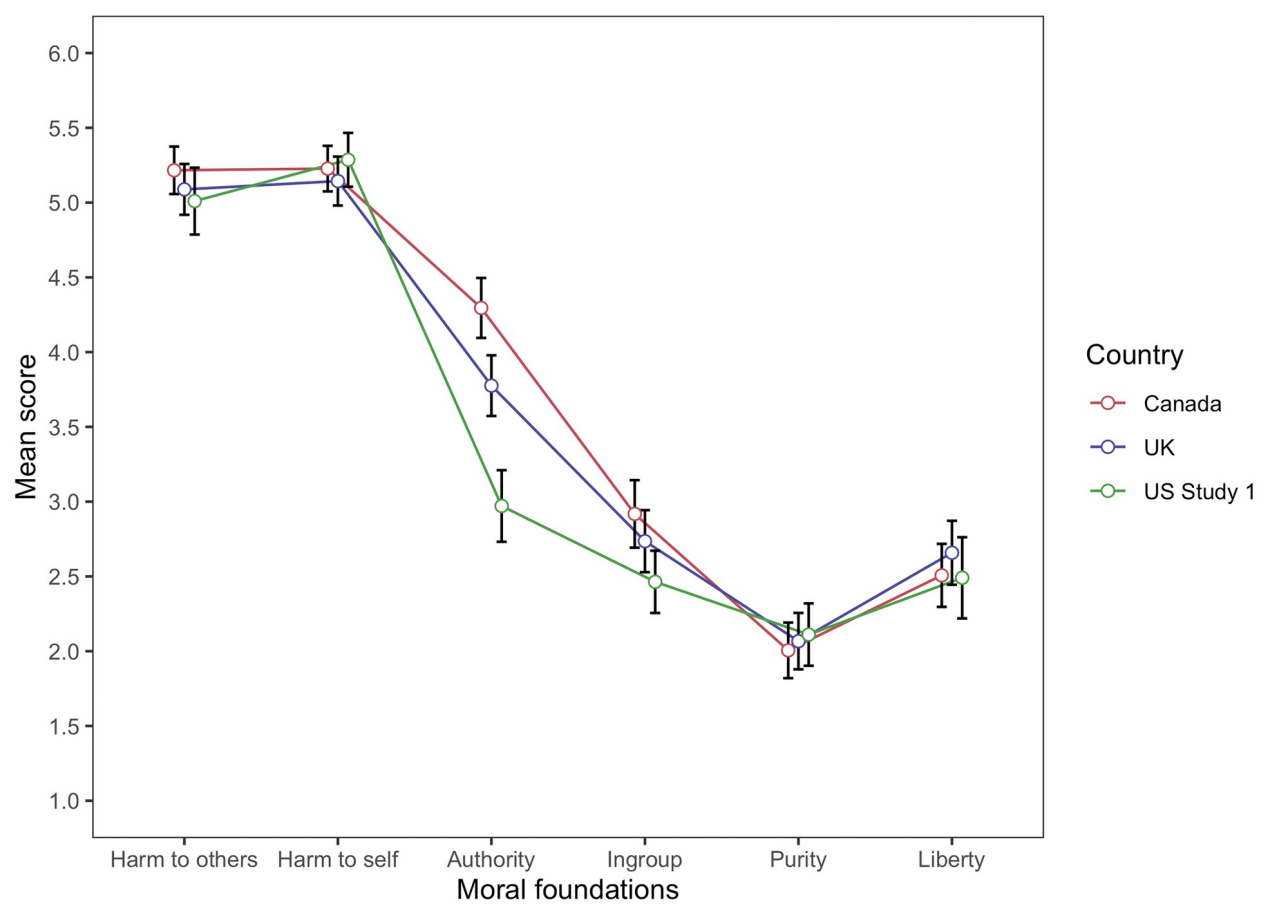
Mean moral foundation scores across the four situations in Study 1. *Note*. The Moral Foundation score ranged from 1 (not relevant) to 6 (extremely relevant).

Average ratings per country for each moral foundation across the four scenarios are plotted in Fig 1. Repeated measures ANOVAs were conducted to assess within-subjects differences in moral foundation scores in each country. Given that Mauchly’s test indicated a violation of sphericity (*p*s < 0.001), degrees of freedom were corrected using Greenhouse-Geiser estimates (US: *ε* = .54; Canada: *ε* = .64; UK: *ε* = .62). We found significant differences in the relative endorsement of different foundations within each country (US: *F*(2.69,368.95) = 179.64, *p* < .001, *η*_p_^2^ = 0.57; Canada: *F*(3.20,560.64) = 257.03, *p* < .001, *η*_p_^2^ = 0.60; UK: *F*(3.12,430.83) = 231.65, *p* < .001, *η*_p_^2^ = 0.63). Posthoc tests with Bonferroni adjustment revealed that in all countries, harm to others and harm to self were rated as more important than the other foundations (*ps* < .001) with the US alone weighing harm to self as more important than harm to others (*p <* .001). In all three countries, the authority foundation then surpassed all other foundations (*ps* < .001), (except for liberty in the US, *p* >.05), with no differences found between ingroup and liberty (*ps* >.05). In Canada and the UK, the purity foundation was rated lower than all other foundations *(ps* < .05), while in the US, it was equivalent to ratings of the ingroup foundation (*p* >.05). Overall, these results suggest that there was a similar pattern of importance attributed to each foundation in the context of COVID-19 situations in each of the three countries. Only the foundation of authority received different importance ratings across the three countries, with the US attributing less importance to it on average.

Spearman’s correlations were conducted to check whether our data showed the kinds of associations between moral foundations and political orientation found in prior research (e.g., Graham et al. [47]) No significant correlations were found in the UK. However, in the US and Canada greater conservatism was related to greater liberty (US: *r*(136) = .39, p < .001; Canada: *r*(174) = .18, p = .02), and purity considerations (US: *r*(136) = .27, p = .001; Canada: *r*(174) = .21, p = .006), but with reduced concern about harm to the self (US: *r*(136) = -.42, p < .001; Canada: *r*(174) = -.29, p < .001), and harm to others (US: *r*(136) = -.44, p < .001; Canada: *r*(174) = -.28, p < .001). These results suggest that, at least in the US and Canada, political ideology was associated with the weight given to different moral foundations, following patterns found in previous research [17].

#### Moral foundations and COVID-19 effective and ineffective behaviors

Consistent with our pre-registration, we conducted separate regressions to explore whether moral foundation scores predicted participants’ engagement in effective and ineffective preventative behaviors in each country. In the presence of outliers, we conducted Linear robust regression [48], and if normality assumptions were violated, we conducted Quantile regression (on the median). We computed the Variance Inflation Factor (VIF) for all the models and found that multicollinearity was not a concern (VIFs were below 3.8). Additionally, as preregistered, countries were not statistically compared given that we could not assume measurement invariance across our intended samples and sample size.

Raw scores indicated that adherence to effective preventative behaviors was relatively high in each country (see Table I in S1 File) and none of the moral foundations significantly predicted engagement in them in either Canada or the UK. However, in the US effective behaviors were positively predicted by greater considerations of harm to others and attention to authority but negatively predicted by concerns about personal liberty (see Table 3).

**Table 3:** Regression of effective and ineffective preventative behaviors on moral foundations in Studies 1 and 2.

	Effective behaviors				Ineffective behaviors			
	Canada	UK	Study 1 US	Study 2 US	Canada	UK	Study 1 US	Study 2 US
Foundations	B (SE)	B (SE)	B (SE)	B (SE)	B (SE)	B (SE)	B (SE)	B (SE)
(Intercept)	-0.62 (0.43)	-1.38 (0.58)*	-2.33 (0.33)	-1.55 (0.37)***	-0.64 (0.37)	-0.61 (0.39)	-1.47 (0.43)***	-0.38 (0.25)
Harm to others	0.12 (0.11)	0.06 (0.09)	0.23 (0.1)*	0.15 (0.07)*	0.09 (0.12)	-0.04 (0.09)	0.1 (0.1)	0.01 (0.05)
Harm to self	0.02 (0.08)	0.21 (0.12)	0.19 (0.11)	0.25 (0.08)**	-0.03 (0.10)	0.07 (0.09)	0.04 (0.1)	0.04 (0.06)
Authority	0.05 (0.04)	0.03 (0.06)	0.06 (0.02)*	-0.01 (0.05)	0.14 (0.07)*	0.14 (0.06)*	0.13 (0.06)*	0.03 (0.04)
Ingroup	0.00 (0.03)	0.01 (0.05)	0.04 (0.03)	0.01 (0.05)	-0.11 (0.05)*	-0.06 (0.06)	0.05 (0.08)	-0.02 (0.04)
Purity	-0.04 (0.04)	-0.01 (0.06)	0.01 (0.03)	-0.01 (0.08)	0.02 (0.06)	0.06 (0.07)	0.12 (0.08)	0.01 (0.05)
Liberty	-0.04 (0.04)	-0.01 (0.05)	-0.07 (0.03)*	-0.14 (0.07)*	-0.01 (0.05)	-0.04 (0.06)	-0.03 (0.06)	-0.03 (0.04)
Regression	Quantile	Quantile	Robust linear	Quantile	Quantile	Quantile	Quantile	Quantile
Adjusted R^2^	.27	.36	.69	.63	.11	.23	.40	.12

Note: For Quantile regression analyses, we report Nagelkerke’s *R*^*2*^. B represents unstandardized regression coefficients.

* *p <* .05,

** *p <* .01,

*** *p <* .001

Regarding ineffective behaviors that have not been an emphasis of prior work, analyses revealed that they were positively predicted by concerns about authority in all countries—a pattern which contrasts that found for effective behaviors. In addition, in Canada alone, ingroup loyalty predicted lower engagement in ineffective behaviors.

Finally, we explored whether political ideology outweighed any of the moral foundations as predictors when added to the model in each country. However, we found that it was not a significant predictor and made no difference to the pattern of results.

#### Moral foundations and intentions to vaccinate against COVID-19

Participants in all three countries tended to express intentions to vaccinate themselves (US: 68%; Canada: 80%; UK: 74%) and their child (US: 62%; Canada: 69%; UK: 63%). To examine the effect of moral foundation scores on intentions to personally vaccinate–an imminent decision–and to vaccinate their child—a hypothetical decision during the study—we conducted two separate logistic regression analyses in each country (see Table 4).

**Table 4:** Regression of intentions to vaccinate the self and the child on moral foundations in Studies 1 and 2.

	Intentions to vaccinate the self				Intentions to vaccinate a child			
	Canada	UK	Study 1 US	Study 2 US^a^	Canada	UK	Study 1 US	Study 2 US
Foundations	OR	OR	OR	OR	OR	OR	OR (95% CI)	OR (95% CI)
	(95% CI)	(95% CI)	(95% CI)	(95% CI)	(95% CI)	(95% CI)		
(Intercept)	0.16	0**	0.06	0***	0.09	0**	0.02* (0.00–0.47)	0** (0.00–0.08)
	(0.01–1.62)	(0.00–0.13)	(0.00–0.90)	(0.00–0.03)	(0.01–0.87)	(0.00–0.10)		
Harm to others	1.44	1.93	1.81	1.51	1.36	1.51	2.1* (1.10–4.37)	1.43 (0.88–2.44)
	(0.69–3.04)	(0.91–4.25)	(0.95–3.68)	(0.94–2.45)	(0.73–2.59)	(0.80–2.87)		
Harm to self	1.54	1.49	1.48	2.52**	1.37	1.53	1.39 (0.61–3.13)	2.34* (1.21–4.86)
	(0.72–3.30)	(0.68–3.26)	(0.68–3.24)	(1.34–5.09)	(0.72–2.62)	(0.80–2.95)		
Authority	1.27	2.42**	1.04	1.62*	1.27	1.67*	1.2 (0.81–1.78)	1.29 (0.94–1.80)
	(0.82–1.99)	(1.41–4.37)	(0.67–1.60)	(1.05–2.57)	(0.90–1.79)	(1.12–2.54)		
Ingroup	2.02*	1.21	1.48	1.66	1.56*	1.25	1.04 (0.65–1.70)	1.19 (0.84–1.70)
	(1.20–3.69)	(0.72–2.11)	(0.88–2.65)	(1.02–2.87)	(1.09–2.31)	(0.86–1.85)		
Purity	0.38***	0.4**	0.46**	0.79	0.5***	0.61*	0.47***(0.29–0.71)	0.65 (0.40–1.03)
	(0.23–0.59)	(0.22–0.69)	(0.28–0.71)	(0.48–1.29)	(0.34–0.70)	(0.38–0.94)		
Liberty	0.59***	0.99	0.75	0.59*	0.74	1.17	0.9 (0.62–1.31)	0.65* (0.43–0.96)
	(0.39–0.86)	(0.61–1.62)	(0.51–1.11)	(0.38–0.91)	(0.54–1.01)	(0.79–1.75)		

Note: OR represents the Odds ratio

^a^ Given the availability of vaccines for adults, in Study 2 this variable includes the participant’s decision to be vaccinated (either with the first dose or fully vaccinated).

* *p <* .05,

** *p <* .01,

*** *p <* .001

Intentions to vaccinate the self and a child were positively predicted by different foundations in each country: attention to authority in the UK, ingroup loyalty in Canada and, for vaccination of a child only, concerns about harm to others in the US. However, purity concerns negatively predicted intentions to vaccinate the self and a child with remarkable consistency in all countries. In Canada, intentions to vaccinate the self were also negatively predicted by concerns about liberty. Again, exploratory analyses found political orientation was not a significant predictor when it was added into models and did not change the pattern of findings.

### Discussion

Consistent with expectations, moral foundations played a significant role in tendencies to engage not only in effective behaviors and intentions to vaccinate but also in ineffective preventative behaviors at the peak of the COVID-19 pandemic. Indeed, the influence of moral foundations outweighed that of political orientation despite prior findings (e.g., Gollwitzer et al. [34]; Pennycook et al. [13]) that the latter have significant effects (see S1 File showing that relationships between political ideology and preventative behavior were often mediated by moral foundations). Patterns in our results revealed cross-country similarities and differences in relationships between moral foundations and preventative behaviors.

#### Cross-country similarities

Participants’ ratings of the importance of each of the foundations were similar in all three countries, with harm (to others or to self) most explicitly weighed when considering pandemic-related situations. It is clear then that people viewed themselves as trying their best to care for themselves and others amidst the pandemic. However, despite being explicitly rated as important, regression analyses revealed that these considerations were not consistently predictive of people’s compliance with effective preventative behaviors. Specifically, while concern about harming others was a significant predictor in the US, it was not predictive in the UK or Canada.

Importantly, two moral foundations consistently predicted health behaviors across all countries. One of these was the authority foundation which—while predicting helpful responses in the UK (intention to vaccinate) and the US (effective behaviors such as social distancing)—was most consistently predictive of *ineffective* behavioral responses. That is, across all countries, there was evidence that attention to public health mandates issued by authorities had the unintended effect of promoting indiscriminate and potentially fearful overreactions, presumably because of participants’ lack of underlying causal knowledge about disease transmission. The fact that strong adherence to mandates increased overreactions, such as complete social isolation, and possibly fear, is worthy of special note given that greater levels of the former are linked to diminished self-regulation skills [27] and greater levels of the latter are associated with heightened depression and anxiety symptoms [49, 50]. Furthermore, the negative effects of overreactions have potential public health system-level effects too insofar as they have the potential to further tax overloaded health care systems by increasing Emergency Department visits [51]. When considered alongside findings that individuals have lower propensities to engage in ineffective preventative behaviors when they have greater biological knowledge [14], these results counsel attention to the unplanned consequences of prescriptive public health messages that are issued—without explanation—in contexts without concerted public education about biology or disease mechanisms.

The second consistent predictor was the purity foundation which, contrary to hypothesis, did not increase ineffective preventative behavior but did have reliably negative predictive effects on intentions to vaccinate the self and children across all countries. This might stem from intuitive perceptions of the vaccine as unnatural and fears that it might taint the body even more than the virus [52]. In the US, these intuitive beliefs were potentially enhanced by presidentially sanctioned scientific misinformation that promoted mistrust in vaccinations—cultural dynamics that were less marked in the UK and Canada [13].

#### Culture-specific patterns

Despite these similarities across all three western countries, culture-specific patterns were also observed. In the UK, the authority foundation had broader predictive effects than in other countries, revealing a particularly marked sense of obligation towards institutions and rules in this country [53]. In Canada, loyalty to the ingroup played a distinctive role, not only predicting increased intentions to vaccinate but reducing engagement in ineffective preventative measures, perhaps because the value placed on the group-binding function of engaging with friends and family acted as a counterweight to overreaching behavioral tendencies that might socially isolate. Finally, there was the unique pattern in the US: Consistent with previous research [10, 23, 24, 32, 54], concerns about harm to others played a distinctive role, predicting increased vaccination intentions and effective preventative behaviors like mask wearing in public spaces. Meanwhile, liberty concerns countervailed—amidst relatively low concerns about authority—to predict reduced engagement in effective behaviors, also predicting reduced vaccination intentions in our other North American country, Canada. As we later discuss, these region-specific patterns may not only reflect subtle differences between the three Western countries in the foundations entrenched in traditions and cultural narratives, but also specific features of the health campaigns and governmental responses at the height of the pandemic. In turn, they therefore also raise a question: how stable are the abstract guiding foundations that predict behavior within a culture when the situational conditions change?

In Study 2, we therefore explored this question at a later time point––the post-peak pandemic period of early summer 2021—in one of the countries. Specifically, we focused on the US during this period because of its rapidly changing context: COVID-19 cases and deaths were dropping [55], mandates in certain states were relaxing, and an increasing number of people returned to work/school in person after the rollout of mass vaccination programs targeting adults and adolescents (which was faster than in other countries). Thus, the US offered a substantial test of whether the predictive effects of moral values are generalizable across contexts. Based on the theorized stability of the moral foundations [15], we hypothesized that Study 2 would find similar patterns as Study 1, except for reduced influences of the authority foundation given reductions in mandates.

## Study 2

### Method

#### Participants

The final sample (N = 170) were U.S. parents of 6-to- 9-year-old children recruited through Prolific who had not participated in prior studies in our labs and had similar demographics to the U.S. sample from Study 1 (see Table 1). The measures for Study 2 were part of a larger survey which aimed to match the sample size of the previous study. Study 2 took place during mid-May and early June 2021, after the peak of the pandemic when death rates were low and approximately 50% of the US population had received their first dose of a COVID-19 vaccine [55]. Participants were excluded if they failed more than one of seven attention checks throughout the survey or at least one of the four attention checks specific to the COVID-19 Moral Foundations Questionnaire (6%). Similar to Study 1, most of the sample (72%) reported having a college degree and leaned liberal politically (M = 2.5, SD = 1.2). All participants provided informed written consent and the survey was approved by Boston University institutional IRB (Approval number: 2350E).

#### Materials and procedures

The measures were the same as in Study 1 with some mild additions and phrasing modifications given the changed context of the pandemic and adult vaccine availability. For instance, one item was about being in a car with friends, but we made clear that the friends were unvaccinated. Additionally, we asked participants to assume–for purposes of answering questions–that they had not yet been vaccinated. See Tables A, C and D in S1 File for items.

In total, we asked about 15 effective and 10 ineffective preventative behavior items. Two composites were created for effective and ineffective behaviors after standardizing each item and obtaining their mean. Given that some participants were fully vaccinated, and others were not for various reasons, we asked them to choose between several options describing their vaccination intentions or decisions. These options were grouped into “yes” and “no” scores to parallel the binary vaccine intention score used in Study 1 (see response options in Tables F and G in S1 File).

In total then, we had four dependent variables: effective behaviors at the post-peak, ineffective behaviors at the post-peak, vaccine intentions for the self and for their child. Additionally, the political orientation scale was made clearer by asking participants to place themselves from “Far left” to “Far right”.

As preregistered, we also obtained participants’ retrospective reports of their behavior at the peak of the pandemic to test the moderation effect of time of data collection (Study 1 vs. Study 2) on the relationship between moral foundations and preventative behaviors. For brevity, we present these analyses in S1 File.

### Results

Cronbach’s alpha revealed acceptable levels of internal consistency in composite scores (behaviors: .62 to .93; moral foundations: .82 to .91 including purity with the same Study 1 item exclusion: .64).

#### Moral foundations across the situations

**Fig 2:**
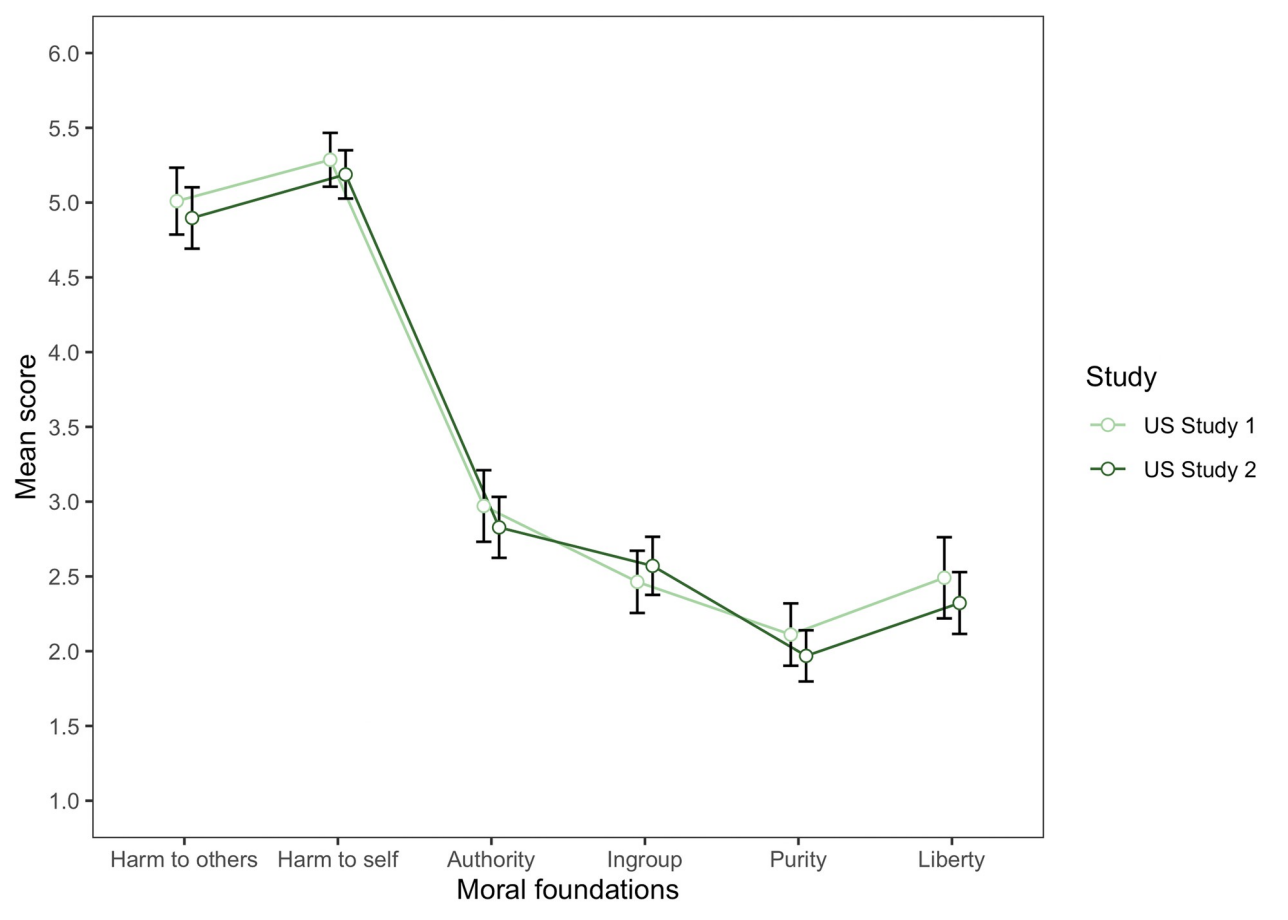
Mean moral foundation scores across the four situations in the US for Studies 1 and 2. *Note*. The Moral Foundation score ranged from 1 (not relevant) to 6 (extremely relevant).

As in Study 1, we conducted a repeated measures ANOVA to assess within-subjects differences in the average score for each moral foundation. Mauchly’s test indicated a violation of sphericity (*p*s < .001), so degrees of freedom were corrected using Greenhouse-Geiser (*ε* = .60). We found significant differences between moral foundations, *F*(2.98,503.99) = 235.62, *p* < .001 (see Fig 2). Posthocs with Bonferroni adjustment revealed similar results to Study 1. U.S. participants tended to weigh harm to self with the greatest relevance (*ps <* .001), followed by harm to others (*ps <* .001). The authority foundation was weighted more strongly than liberty and purity (*ps <* .05), no differences were found between liberty and ingroup, but the purity foundation (*ps* < .001) was weighted lower than other foundations.

Spearman’s correlations revealed that, consistent with Study 1, greater conservatism was related to greater considerations of purity (*r*(168) = 0.34, *p <* .001), and liberty (*r*(168) = 0.44, *p <* .001), but to lower considerations of harm to others (*r*(168) = -0.35, *p <* .001) or self, (*r*(168) = -0.38, *p <* .001).

#### Moral foundations and effective and ineffective behaviors at post-peak

Due to normality violations, quantile regressions were conducted to test the effects of the moral foundations on effective and ineffective preventative behaviors. Additionally, we computed the Variance Inflation Factor (VIF) for all the models below and found that multicollinearity was not a concern (VIFs were below 2.95). As with U.S. participants in Study 1, we found that once again, concerns about harm to others positively predicted effective preventative behaviors in the US while liberty concerns negatively predicted them. In a new pattern, concerns about harm to self were significantly associated with greater engagement in effective behaviors but as predicted given relaxing mandates, concerns about authority were no longer predictive (see Table 3). Interestingly, and in contrast to Study 1, the authority foundation was no longer predictive of ineffective behaviors and no other foundations were significant either.

Exploratory analyses found political orientation did not significantly predict when it was added into models and did not change findings for ineffective behaviors. Meanwhile, for effective behaviors, although political ideology remained non-significant, harm to others and the liberty foundation stopped being significant—likely because of the high correlation between liberty and conservatism, *r*(168) = .44, p < .001.

#### Moral foundations and intentions to vaccinate against COVID-19 at post-peak

In Study 2, 71% participants expressed pro-vaccination attitudes for themselves (61% were partially or fully vaccinated). However, 50% of this parent sample indicated that they did *not* intend to vaccinate their child when federally approved vaccinations became available. Participants’ intentions to get themselves or their child vaccinated were positively predicted by concerns about harm to self, and negatively predicted by liberty. In contrast with Study 1, in which purity had a significant effect, purity was no longer predictive of intentions to vaccinate (self or child). Moreover, the authority foundation only positively predicted intentions to vaccinate the self.

Finally, political orientation was not a significant predictor when it was added into each model but again, when it was added in, the liberty foundation lost significance presumably because of its high correlation with conservatism.

### Discussion

In Study 2, we explored the stability of moral foundations as predictors of preventative behaviors by collecting a second US sample approximately 6 months after we collected the data described in Study 1. In general, we found that patterns of results at Time 2 largely mirrored those at Time 1, especially those related to engagement in effective preventative behaviors.

#### Harm to self and others

As in Study 1, U.S. participants rated harm as by far the most important moral foundation, and also rated harm to the self as more important than harm to others. Likewise, as in Study 1, considerations of harm were the strongest predictors of effective preventative behaviors, although contrary to Study 1, both harm to the self and harm to others predicted effective preventative behaviors. Indeed, by Study 2, considerations of harm to self, rather than harm to others, predicted vaccination intentions for both participants themselves and their children. These findings highlight the importance of concerns about harm within a liberal leaning U.S. sample [17]. However, they suggest that once the greatest risk of serious illness and death passes, U.S. participants become focused on personal risk when making health decisions. The broad implication of this is that public health communicators in the US should emphasize the benefits of preventative behaviors for the self, not the whole population, once a disease outbreak has peaked.

#### Authority

As in Study 1, authority was the next most important moral foundation for the US sample and remained an important predictor of health behaviors but not in the same way as in Study 1. In line with our hypothesis, by Study 2 authority no longer predicted either effective or ineffective behaviors, perhaps because public health mandates were being relaxed and public health communications were becoming less salient. However, unlike Study 1, authority did positively predict intention to get vaccinated. This result is potentially explained by the fact that, by the pandemic post-peak, many institutions (e.g., airlines) were considering mandating vaccination [56].

#### Ingroup, purity

Considerations of ingroup and purity were unrelated to effective or ineffective behaviors or to vaccination intentions. This represents a departure; purity concerns predicted vaccination intentions in Study 1, but no longer did so by the time of Study 2. One explanation for this unexpected result, is that by the post-peak, as vaccines became mandatory, it had become clear that vaccines were safe, and so participants were more concerned about vaccines as a violation of liberty than as a violation of purity.

#### Liberty

As in Study 1, liberty continued to be negatively associated with effective preventative behaviors. Unlike Study 1, liberty—rather than purity—negatively predicted vaccination intentions for both self and child. These findings confirm that liberty is a robust value in North American health decision-making—one that relates to vaccine hesitancy and is prominent among conservatives [25, 32].

#### Summary

In large part, patterns in Study 2 largely mirrored US patterns observed in Study 1. Indeed, as additional evidence of this, analyses found that data collection time-point (peak vs. post-peak) had no moderating effect on the relationship between moral foundations and health behaviors (see S1 File). Together, these findings provide support for the hypothesis that the effects of moral foundations are largely stable across time within a culture, and therefore have the potential to exert enduring influences on decisions [16].

## General discussion

Prior research has suggested that moral foundations relate to health decision-making (e.g., Amin et al., [25]; Chan, [10]; Pagliaro et al., [21]). However, such studies have often been limited to singular time points and have not differentiated out the specific effective or ineffective nature of the health behaviors. In the present two studies, we addressed those gaps in the literature and confirmed that in the context of a global pandemic, moral foundations influenced people’s engagement in not only effective but also ineffective preventative behaviors across countries and time. These findings have important implications for health communication and interventions.

### Cross-country similarities

Specifically, and with strong relevance to public policy, we found that in Canada, the US, and the UK, people’s tendencies to attend to and respect authority had expected as well as unexpected effects. While concerns about authority and official recommendations seemed to promote effective behaviors in most countries, including in the US across time points, they also showed signs of having unintentional consequences by promoting ineffective responses across countries too. Given that ineffective behavioral responses are not truly protective—but can lead to a false sense of security—and can also diminish quality of life when they become fearful overreactions [27, 49, 50], the factors that promote them are worthy of attention and have implications for health communication. Given the risk of unintended consequences, our findings suggest that any institutional messaging about health guidelines should be performed cautiously and be accompanied by educational materials that explain disease transmission processes so that appropriate effective behaviors are promoted. Importantly, such materials should also inform people why certain behaviors are an ineffective overreach. This recommendation is further bolstered by research demonstrating that factual knowledge about the coronavirus is associated with positive health behaviors [35], while an absence of general biological knowledge relates to ineffective ones [14].

Purity also played an important role in all three countries. Consistent with prior research [25, 33], and with our own expectations we found that, across cultures, purity concerns reduced intentions to vaccinate during the pandemic peak, presumably due to perceptions of the vaccine as unnatural—concerns likely enhanced for mRNA vaccines given widespread misinformation that they alter human DNA [57, 58]. Contrary to expectations, however, purity did not increase either ineffective or effective behavioral responses, even as purity has been proposed as the outgrowth of an adaptation for disease avoidance [18]. This finding is consistent with more recent research that cumulatively suggests that the purity foundation is an inconsistent influence on health behavior (see Qian & Yahara [22]; Rosenfeld & Tomiyama [54]; Tarry et al. [32]). Further work is needed to fully understand the specific contexts that lead the purity foundation to have an influence on health behaviors. Two possibilities seem worthy of future exploration. First, it is possible that this pattern is tied to the way disease features are described, with “airborne diseases” specifically failing to consistently elicit purity considerations because their air-based mode of transmission induces less disgust. It remains an open question therefore whether diseases with other kinds of features (e.g., sexually-transmitted diseases) elicit purity concerns more strongly. Second, it is possible that people who weigh purity considerations might perceive the virus as more natural relative to a vaccine that is human-made. Further research is needed to test whether different narrative framings of vaccination might change this perception.

In addition to these findings across cultures, our research also shed light on more general dynamics—patterns that contrast with a large body of research emphasizing the effect of political ideologies on health behaviors. Specifically, our data indicate that the effects of moral foundations can enduringly outweigh those of political ideology. Indeed, some foundations appear to operate as the mechanism through which political ideology impacts specific behaviors (see S1 File), as hinted at in related recent research [23, 32]. These findings also have implications for health interventions. Specifically, they suggest that rather than targeting political affiliations [59], health interventions might gain in effectiveness by speaking to a range of moral values such as considerations of authority and purity.

### Culture-specific patterns

Apart from similarities across countries, our research also revealed nuanced culture-specific patterns. In the UK, it was noteworthy that participants weighed authority more broadly. Attention to authority not only predicted ineffective behaviors, as in the other countries, and effective behaviors, as in some, but extended to vaccination intentions at a time when vaccines were not even available or mandated. One explanation for this pattern is that, historically, British culture has not only been hierarchical (e.g., social class structures), but focused on respect for laws and venerable institutions [53, 60]. Indeed, public health campaigns seemed to leverage this fact. For example, the National Health System (NHS) is a source of British pride, and this is presumably why UK health campaigns underscored the importance of avoiding NHS collapse rather than focusing on individual protection [61]. Also, participants with stronger national identity may have felt more motivated to receive the COVID-19 vaccine given its publicly-funded development by Oxford University.

Another notable culture-specific pattern occurred in Canada where participants placed high value on loyalty to their community. Specifically, Canadians’ emphasis on the ingroup foundation prevented them from engaging in overreactive ineffective behaviors, and also predicted higher motivation to get the vaccine. Again, there was some evidence that Canadian pandemic health campaigns aligned to—and maybe even strengthened—the culture-specific value of loyalty to community. For example, various campaigns referred to the “Ripple Effect” and accentuated individual responsibility for the health future of the whole community [62].

By contrast to the other countries, the US demonstrated some singularity given the degree to which the harm and liberty foundations had predictive power. In the case of considerations about harm, either directed to the self or others, it was a strong predictor of preventative behaviors, including intentions to vaccinate, which is consistent with the prior research [10]. Unlike in our other countries, not only considerations of authority were not given a high relevance, but also liberty considerations predicted less compliance with effective preventative behaviors. By Study 2, this foundation was also predictive of lower intentions to vaccinate the self or the child (a pattern limited to Canada in Study 1). Given that libertarians tend to be individualistic, and perceive health mandates as oppressive, persuading them to engage in behavioral change by appealing to the collective benefits of vaccination, or the behavioral standards set by political elites [63], is unlikely to be effective. Research should therefore explore whether messages might be more effective if they emphasize good health as prerequisite to exercising personal freedom.

### Stability over time

In the US, the rating of importance given to each of the foundations remained the same in both studies which is consistent with the notion that moral foundations represent robust culture-specific influences on decision-making and are generally stable [16].

Additionally, regression analyses also suggested stability across time: the time point of data collection did not moderate the effect of moral foundations on health behaviors. Although some foundations, such as purity and authority, stopped being predictive of preventative behaviors—probably due to greater accessibility to information about the virus and the vaccines, as well as the loosening of public health regulations—the foundations of harm and liberty continued to have a very significant effect. With respect to harm, our contextualized measure of moral foundations was effective in identifying that effective behaviors (including vaccination) were positively predicted by considerations about harm to others at the peak, and to the self at post-peak. These findings extend prior work demonstrating strong links between health decision-making in the US and concerns about avoidance of harm [10], by showing that the locus of that predictive concern subtly shifts with context. Finally, the negative effect of liberty considerations on US participants’ adherence to effective health behaviors remained stable across time—just as persistent lockdown protests might suggest [64]—which once again confirms the important role of this value in U.S. society [19].

One reason we detected some changes in the predictive relationship between some moral foundations and protective behaviors when comparing our two time points may be that we used a contextualized version of the MFQ rather than the traditional MFQ. Although using a contextualized MFQ allowed us to test the specific foundations that participants rated as relevant in Covid-related situations, this measure may also have been more sensitive to subtle changes in situational factors. Our measure, therefore, contrasts with the traditional MFQ that can be generalizable to a variety of contexts. Specifically, the pandemic circumstances were not only novel and unexpected but also constantly transforming. As time passed, more considerations started to play a role in participants’ preventative behaviors. For instance, the growing availability of vaccines, changes in the perceived severity of the virus, or changes in government mandates might all have been contextual factors that changed the predictive significance of some foundations over time. Our contextualized measure potentially offered a higher level of sensitivity in detecting these dynamics. Further research comparing predictive patterns at different time points during a pandemic (e.g., pandemic peak and post-pandemic periods) with the MFQ and our contextualized MFQ will clarify whether this supposition of increased sensitivity proves correct.

### Limitations and further research

There are two main limitations in this study that could be addressed in further research. First, we deliberately focused our study on parents. It is possible that parents’ moral concerns differ systematically from those of non-parents, and that the patterns we observed here may represent a special case. We think this is unlikely because our results show a good deal of consistency with prior research on effective preventative behaviors that was not focused on parents (e.g., like multiple studies [10, 21, 32, 65], we found that endorsement of the individualizing foundations is predictive of greater preventative behaviors in the US). Nevertheless, we were not able to directly test whether our results broadly generalize. Therefore, further research should explore whether specific groups within a population (e.g., parents, health workers, adolescents, etc.)—which might be more or less used to prioritizing their responsibilities to others—tend to perceive some foundations as more relevant than others and whether that impacts their health behaviors in different ways.

Second, although we find an important effect of moral foundations on health behaviors that outweighed effects of political ideology, further experimental research is needed to confirm a causal relationship and identify underlying mechanisms. Indeed, research in the future should explore whether campaigns using messages that highlight specific moral considerations are more effective than those campaigns that do not emphasize them or emphasize political ideology and disease severity instead.

## Conclusions

In sum, our results show that, as stable influences within a culture, moral values should be an important focus when designing campaigns that not only promote effective health behaviors but importantly prevent people from engaging in ineffective behaviors. This is especially relevant given that pandemics are likely to recur increasingly in the future and preventing ineffective behaviors might reduce the associated negative effects on psychological wellbeing and on the health system. Research has already demonstrated the effectiveness of communicating messages that match recipient-endorsed moral beliefs [66, 67]. Our data support that such strategies are likely to have greater impact longer-term than focusing on political affiliations especially if they are accompanied by accurate biological information explaining why certain behaviors are effective or ineffective. Meanwhile interventions focused on decreasing or redirecting liberty and purity concerns seem crucial to reducing vaccination hesitancy. Given that our participants were parents, our findings are especially relevant to the design of family education programs that impact future generations’ health decisions.

## Supporting information

S1 File (DOCX)

## References

[1] Ali SH, Keil R. Global Cities and the Spread of Infectious Disease: The Case of Severe Acute Respiratory Syndrome (SARS) in Toronto, Canada: Urban Stud 2016;43:491–509. doi: 10.1080/00420980500452458

[2] World Health Organization. Global Crises—Global Solutions Managing public health emergencies of international concern through the revised International Health Regulations. WHO 2002.

[3] Fidler DP. Germs, governance, and global public health in the wake of SARS. J Clin Invest 2004;113:799–804. doi: 10.1172/JCI21328 15067309 PMC362129

[4] O’Connor DB, Aggleton JP, Chakrabarti B, Cooper CL, Creswell C, Dunsmuir S, et al. Research priorities for the COVID-19 pandemic and beyond: A call to action for psychological science. Br J Psychol 2020;111:e12468. doi: 10.1111/bjop.12468 32683689 PMC7404603

[5] Taylor S. The psychology of pandemics: preparing for the next global outbreak of infectious disease. England: Cambridge Scholars Publishing.; 2019. doi: 10.1080/03069885.2021.1949809

[6] Weston D, Hauck K, Amlôt R. Infection prevention behaviour and infectious disease modelling: A review of the literature and recommendations for the future. BMC Public Health 2018;18:1–16. doi: 10.1186/s12889-018-5223-1 29523125 PMC5845221

[7] Fazio RH, Ruisch BC, Moore CA, Granados Samayoa JA, Boggs ST, Ladanyi JT. Who is (not) complying with the U. S. social distancing directive and why? Testing a general framework of compliance with virtual measures of social distancing. PLOS ONE 2021;16. doi: 10.1371/journal.pone.0247520 33626066 PMC7904183

[8] Greene CM, Murphy G. Quantifying the effects of fake news on behavior: Evidence from a study of COVID-19 misinformation. J Exp Psychol Appl 2021;27:773–84. doi: 10.1037/xap0000371 34110860

[9] Byrd N, Białek M. Your health vs. my liberty: Philosophical beliefs dominated reflection and identifiable victim effects when predicting public health recommendation compliance during the COVID-19 pandemic. Cognition 2021;212. doi: 10.1016/j.cognition.2021.104649 33756152 PMC8599940

[10] Chan EY. Moral foundations underlying behavioral compliance during the COVID-19 pandemic. Personal Individ Differ 2021;171. doi: 10.1016/j.paid.2020.110463 33106715 PMC7577686

[11] Haidt J. The emotional dog and its rational tail: A social intuitionist approach to moral judgment. Reason. Stud. Hum. Inference Its Found., New York, US: Cambridge University Press; 2008, p. 1024–52. doi: 10.1017/CBO9780511814273.055

[12] Godin G, Conner M, Sheeran P. Bridging the intention–behaviour gap: The role of moral norm. Br J Soc Psychol 2005;44:497–512. doi: 10.1348/014466604X17452 16368016

[13] Pennycook G, McPhetres J, Bago B, Rand DG. Beliefs About COVID-19 in Canada, the United Kingdom, and the United States: A Novel Test of Political Polarization and Motivated Reasoning. Pers Soc Psychol Bull 2021:014616722110236. doi: 10.1177/01461672211023652 34180276 PMC9066691

[14] Ronfard et al. What predicts adults’ selective implementation of COVID-19 preventative measures? Data from the United States, Canada, and the United Kingdom. Manuscript in preparation. n.d.

[15] Haidt J, Joseph C. Intuitive ethics: how innately prepared intuitions generate culturally variable virtues. Daedalus 2004;133:55–66. doi: 10.1162/0011526042365555

[16] Graham J, Haidt J, Koleva S, Motyl M, Iyer R, Wojcik SP, et al. Moral Foundations Theory: The Pragmatic Validity of Moral Pluralism. Adv Exp Soc Psychol 2013;47. doi: 10.1016/B978-0-12-407236-7.00002-4

[17] Haidt J, Graham J. When morality opposes justice: Conservatives have moral intuitions that liberals may not recognize. Soc Justice Res 2007;20:98–116. doi: 10.1007/s11211-007-0034-z

[18] Haidt J. The moral foundations of politics. Righteous Mind Why Good People Are Divid. Polit. Relig., New York: Pantheon Books; 2012.

[19] Iyer R, Koleva S, Graham J, Ditto P, Haidt J. Understanding libertarian morality: The psychological dispositions of self-identified libertarians. PLOS ONE 2012;7. doi: 10.1371/journal.pone.0042366 22927928 PMC3424229

[20] Lo Presti S, Mattavelli G, Canessa N, Gianelli C. Psychological precursors of individual differences in COVID-19 lockdown adherence: Moderated-moderation by personality and moral cognition measures. Personal Individ Differ 2021;182:111090. doi: 10.1016/j.paid.2021.111090 36540872 PMC9756798

[21] Pagliaro S, Sacchi S, Pacilli MG, Brambilla M, Lionetti F, Bettache K, et al. Trust predicts COVID-19 prescribed and discretionary behavioral intentions in 23 countries. PLOS ONE 2021;16:1–16. doi: 10.1371/journal.pone.0248334 33690672 PMC7946319

[22] Qian K, Yahara T. Mentality and behavior in COVID-19 emergency status in Japan: Influence of personality, morality and ideology. PLOS ONE 2020;15:1–16. doi: 10.1371/journal.pone.0235883 32649687 PMC7351180

[23] Bruchmann K, LaPierre L. Moral Foundations Predict Perceptions of Moral Permissibility of COVID-19 Public Health Guideline Violations in United States University Students. Front Psychol 2022;12:795278. doi: 10.3389/fpsyg.2021.795278 35185693 PMC8847225

[24] Reimer NK, Atari M, Karimi-Malekabadi F, Trager J, Kennedy B, Graham J, et al. Moral Values Predict County-Level COVID-19 Vaccination Rates in the United States [preprint in osf.io] n.d. 10.1037/amp0001020 36074569

[25] Amin AB, Bednarczyk RA, Ray CE, Melchiori KJ, Graham J, Huntsinger JR, et al. Association of moral values with vaccine hesitancy. Nat Hum Behav 2017;1:873–80. doi: 10.1038/s41562-017-0256-5 31024188

[26] Graham J, Nosek BA, Haidt J, Iyer R, Koleva S, Ditto PH. Mapping the Moral Domain. J Pers Soc Psychol 2011;101:366–85. doi: 10.1037/a0021847 21244182 PMC3116962

[27] Hawkley LC, Cacioppo JT. Loneliness Matters: A Theoretical and Empirical Review of Consequences and Mechanisms. Ann Behav Med 2010;40:218–27. doi: 10.1007/s12160-010-9210-8 20652462 PMC3874845

[28] Rossen I, Hurlstone MJ, Dunlop PD, Lawrence C. Accepters, fence sitters, or rejecters: Moral profiles of vaccination attitudes. Soc Sci Med 2019;224:23–7. doi: 10.1016/j.socscimed.2019.01.038 30735925

[29] Demerath NJ. Excepting Exceptionalism: American Religion in Comparative Relief. vol. 558. 1998.

[30] Nolan E, Kennedy M. Explaining the American Exceptionalism. INOSR Arts Humanit 2017;3:1–8.

[31] Harper CA, Satchell LP, Fido D, Latzman RD. Functional Fear Predicts Public Health Compliance in the COVID-19 Pandemic. Int J Ment Health Addict 2020. doi: 10.1007/s11469-020-00281-5 32346359 PMC7185265

[32] Tarry H, Vézina V, Bailey J, Lopes L. Political orientation, moral foundations, and COVID-19 social distancing. PLOS ONE 2022;17:e0267136. doi: 10.1371/journal.pone.0267136 35749535 PMC9232135

[33] Schmidtke KA, Kudrna L, Noufaily A, Stallard N, Skrybant M, Russell S, et al. Evaluating the relationship between moral values and vaccine hesitancy in Great Britain during the COVID-19 pandemic: A cross-sectional survey. Soc Sci Med 2022;308:115218. doi: 10.1016/j.socscimed.2022.115218 35870299 PMC9281411

[34] Gollwitzer A, Martel C, Brady WJ, Pärnamets P, Freedman IG, Knowles ED, et al. Partisan differences in physical distancing are linked to health outcomes during the COVID-19 pandemic. Nat Hum Behav 2020;4:1186–97. doi: 10.1038/s41562-020-00977-7 33139897

[35] Moore CA, Ruisch BC, Granados Samayoa JA, Boggs ST, Ladanyi JT, Fazio RH. Contracting COVID-19: a longitudinal investigation of the impact of beliefs and knowledge. Sci Rep 2021;11:1–12. doi: 10.1038/s41598-021-99981-8 34650222 PMC8516850

[36] Stroebe W, vanDellen MR, Abakoumkin G, Lemay EP, Schiavone WM, Agostini M, et al. Politicization of COVID-19 health-protective behaviors in the United States: Longitudinal and cross-national evidence. PLOS ONE 2021;16:e0256740. doi: 10.1371/journal.pone.0256740 34669724 PMC8528320

[37] Merkley E, Bridgman A, Loewen PJ, Owen T, Ruths D, Zhilin O. A rare moment of cross-partisan consensus: Elite and public response to the CoviD-19 pandemic in Canada. Can J Polit Sci 2020;53:311–8. doi: 10.1017/S0008423920000311

[38] Van Leeuwen F, Park JH, Koenig BL, Graham J. Regional variation in pathogen prevalence predicts endorsement of group-focused moral concerns. Evol Hum Behav 2012;33:429–37. doi: 10.1016/j.evolhumbehav.2011.12.005

[39] WHO. COVID-19 Weekly Epidemiological Update Global summary. 2020.

[40] FDA. FDA Takes Additional Action in Fight Against COVID-19 By Issuing Emergency Use Authorization for Second COVID-19 Vaccine. FDA News Release 2020:1–5.

[41] WHO. WHO issues its first emergency use validation for a COVID-19 vaccine and emphasizes need for equitable global access. WHO 2020:1–3.

[42] COVID-19 Treatment Guidelines Panel. Coronavirus Disease 2019 (COVID-19) Treatment Guidelines May 2021. National Institutes of Health. 2021. https://www.covid19treatmentguidelines.nih.gov/. (accessed February 3, 2022). 34003615

[43] Honein MA, Christie A, Rose DA, Brooks JT, Meaney-Delman D, Cohn A, et al. Morbidity and Mortality Weekly Report Summary of Guidance for Public Health Strategies to Address High Levels of Community Transmission of SARS-CoV-2 and Related Deaths, December 2020. Morb Mortal Wkly Rep 2020;69:1860–7. 10.15585/mmwr.mm6949e2 PMC7737690 33301434

[44] WHO. Overview of public health and social measures in the context of COVID-19. Interim guidance 18 May 2020. 2020.

[45] Grant AM, Hofmann DA. It’s Not All About Me: Motivating Hand Hygiene Among Health Care Professionals by Focusing on Patients. Psychol Sci 2011;22:1494–9. doi: 10.1177/0956797611419172 22075239

[46] Probst S, Nowack A, Warneken F. Children’s moral reasoning about self- versus other-benefiting public health measures. J Exp Child Psychol 2023;229:105623. doi: 10.1016/j.jecp.2022.105623 36696739 PMC9868488

[47] Graham J, Haidt J, Nosek BA. Liberals and Conservatives Rely on Different Sets of Moral Foundations. J Pers Soc Psychol 2009;96:1029–46. doi: 10.1037/a0015141 19379034

[48] Berk RA. A primer on robust regression. Mod Methods Data Anal 1990:292–324.

[49] Brooks SK, Webster RK, Smith LE, Woodland L, Wessely S, Greenberg N, et al. The psychological impact of quarantine and how to reduce it: rapid review of the evidence. The Lancet 2020;395:912–20. doi: 10.1016/S0140-6736(20)30460-8 32112714 PMC7158942

[50] Fitzpatrick KM, Drawve G, Harris C. Facing new fears during the COVID-19 pandemic: The State of America’s mental health. J Anxiety Disord 2020;75:102291. doi: 10.1016/j.janxdis.2020.102291 32827869 PMC7425672

[51] McDonnell WM, Nelson DS, Schunk JE. Should we fear “flu fear” itself? Effects of H1N1 influenza fear on ED use. Am J Emerg Med 2012;30:275–82. doi: 10.1016/j.ajem.2010.11.027 21208765

[52] Powell D, Weisman K, Markman EM. Articulating lay theories through graphical models: A study of beliefs surrounding vaccination decisions. CogSci, 2018, p. 906–11.

[53] Park A, Curtice J, Thomson K, Bromley C. British Social Attitudes: The 21st Report. London: SAGE Publications; 2004. doi: 10.4135/9781849208666

[54] Rosenfeld DL, Tomiyama AJ. Moral Judgments of COVID-19 Social Distancing Violations: The Roles of Perceived Harm and Impurity. Pers Soc Psychol Bull 2021. doi: 10.1177/01461672211025433 34247528

[55] CDC. What’s up, Doc? COVID Data Tracker Wkly Rev May 28th 2021:1–7.

[56] Diamond D, Sun LH, Stanley-Becker I. ‘Vaccine passports’ are on the way, but developing them won’t be easy. Wash Post 2021:4.

[57] Carmichael F, Goodman J. Vaccine rumours debunked: Microchips, “altered DNA” and more. BBC News 2020.

[58] CDC. Myths and Facts about COVID-19 Vaccines. Cent Dis Control Prev 2022. https://www.cdc.gov/coronavirus/2019-ncov/vaccines/facts.html (accessed June 16, 2022).

[59] Haidt J, Graham J, Joseph C. Above and below left-right: Ideological narratives and moral foundations. Psychol Inq 2009;20:110–9. doi: 10.1080/10478400903028573

[60] More in Common. Shared Identity [Chapter 7]. Britain’s Choice Common Ground Div. 2020s Br., 2020, p. 2–21.

[61] Department of Health and Social Care. New TV advert urges public to stay at home to protect the NHS and save lives—GOV.UK 2021. https://www.gov.uk/government/news/new-tv-advert-urges-public-to-stay-at-home-to-protect-the-nhs-and-save-lives (accessed December 7, 2021).

[62] Public Health Agency of Canada. Government of Canada launches new “Ripple Effect” advertising campaign to encourage COVID-19 vaccination 2021. https://www.canada.ca/en/public-health/news/2021/05/government-of-canada-launches-new-ripple-effect-advertising-campaign-to-encourage-covid-19-vaccination.html (accessed December 7, 2021).

[63] Pink SL, Chu J, Druckman JN, Rand DG, Willer R. Elite party cues increase vaccination intentions among Republicans. Proc Natl Acad Sci 2021;118:1–3. doi: 10.1073/pnas.2106559118 34312254 PMC8364165

[64] Andone D. Protests are popping up across the US over stay-at-home restrictions—CNN. CNN 2020.

[65] Nan X, Wang Y, Thier K, Adebamowo C, Quinn S, Ntiri S. Moral Foundations Predict COVID-19 Vaccine Hesitancy: Evidence from a National Survey of Black Americans. J Health Commun 2022;27:801–11. doi: 10.1080/10810730.2022.2160526 36576158

[66] Trevors G, Duffy MC. Correcting COVID-19 Misconceptions Requires Caution. Educ Res 2020;49:538–42. doi: 10.3102/0013189X20953825

[67] Voelkel JG, Feinberg M. Morally Reframed Arguments Can Affect Support for Political Candidates. Soc Psychol Personal Sci 2018;9:917–24. doi: 10.1177/1948550617729408 30595808 PMC6295651

