# Supplementary material for: Why we should care about moral foundations when preparing for the next pandemic: Insights from Canada, the UK and the US
Source: PLoS One. 2023 May 12;18(5):e0285549. doi: 10.1371/journal.pone.0285549 (PMC10180656; doi:10.1371/journal.pone.0285549)
Supplement: S1 File — (DOCX) [file pone.0285549.s001.docx]

**Supplementary Information**

Table of Contents

[Table of Contents 1](#_Toc128301538)

[Materials Study 1 2](#_Toc128301539)

[Table A. COVID-19 Engagement in Effective and Ineffective Behaviors Questionnaire Items (Study 1) 2](#_Toc128301540)

[COVID-19 Moral Foundations Questionnaire – Hypothetical Scenarios 3](#_Toc128301541)

[Table B. Moral Foundations Questionnaire Items in Each Scenario 4](#_Toc128301542)

[Materials Study 2 4](#_Toc128301543)

[Table C. New Items Added to the COVID-19 Engagement in Preventative Effective Behaviors Questionnaire (Study 2) 5](#_Toc128301544)

[Table D. Modified Items of the COVID-19 Engagement in Preventative Effective and Ineffective Behaviors Questionnaire for Study 2 5](#_Toc128301545)

[Table E. Items in Moderation Analysis from Study 1 (Peak in December) and Study 2 (Post-peak in May) 6](#_Toc128301546)

[Table F. Score Assigned to Choices about Intention to Vaccinate the Self 7](#_Toc128301547)

[Table G. Score Assigned to Choices about Intention to Vaccinate their Child 8](#_Toc128301548)

[Supplementary Results 9](#_Toc128301549)

[Table H. Mean Moral Foundation Score by Scenario and Country 9](#_Toc128301550)

[Table I. Average Effective Preventative Behaviors in Study 1 and Study 2 (raw scores per item) 10](#_Toc128301551)

[Table J. Average Ineffective Preventative Behaviors in Study 1 and Study 2 (Raw Scores per Item) 11](#_Toc128301552)

[Stability of the Effect of the Moral Foundations across Time Points 12](#_Toc128301553)

[Predictors of Change in Preventative Behaviors Between Peak and Post-peak 12](#_Toc128301554)

[Is the Effect of Political Ideology on Preventative Behaviors Mediated by Moral Foundations? 13](#_Toc128301555)

[Table K. Demographics of Indian Sample 17](#_Toc128301556)

[Table L. Mean Moral Foundation Score by Scenario in India 17](#_Toc128301557)

[Table M. Regression of Dependent Variables on Moral Foundations in India 18](#_Toc128301558)

Materials Study 1

Table A. COVID-19 Engagement in Effective and Ineffective Behaviors Questionnaire Items (Study 1)

| Type of behavior | Item |
| --- | --- |
| **Effective preventative behaviors** | |
| Wearing a mask ^a^ | When grocery shopping |
|  | When taking public transportation |
|  | When in a car with people I don’t live with |
|  | When in school |
| Social distancing (reverse coded) ^b^ | Shake other people’s hands |
|  | Go to indoor restaurants |
|  | Go to indoor bars |
|  | Attend events with 10 or more people indoors |
| Washing ^b^ | Avoid touching my face |
|  | Wash my hands for 20 seconds |
| Removed items | Go to outdoor restaurants |
|  | Wash groceries after shopping |
|  | Require my child to use hand sanitizer |
| **Ineffective preventative behaviors** | |
| Wearing a mask ^a^ | When I am outdoors alone |
|  | When outdoors around other people but able to stay six feet away |
|  | When socializing with people I live with |
| Social distancing (reverse coded) ^b^ | Borrow tools/objects from my neighbor |
|  | Feed my friend's pet when they are not there |
|  | Exercise outdoors when I am unlikely to encounter other people |
| Washing ^b^ | Use special “antibacterial” soap |
|  | Wash my children’s clothes |
|  | Have my child bathe/shower |
|  | Purify the air in my house |
| Removed item | Pet a stranger's dog |

^a^ Participants answered *yes* or *not* to questions about whether they would wear a mask in the situations described in each item. ^b^ Participants rated the frequency of engagement in the item behaviors (social-distancing and washing) relative to pre-pandemic times from 1 = *much less frequently* to 5 = *much more frequently*.

COVID-19 Moral Foundations Questionnaire – Hypothetical Scenarios

Participants were presented with four hypothetical scenarios described below and Table B shows the seven items presented with each scenario. Participants rated each foundation item on a scale from 1 = *not at all relevant* to 6 = *extremely relevant*.

**Mask Wearing Scenario.** One of your friends invited you to a party with a bunch of people. When you decide whether or not to wear a mask, to what extent are the following considerations likely to be relevant to your thinking?

**Social Distancing Scenario.** A friend travelled on a long-haul flight and a few days after landing comes to visit you at your home. When you decide whether or not to keep social distance (stay at least 6 feet away) from your friend, to what extent are the following considerations likely to be relevant to your thinking?

**Intentions to Personally Vaccinate Scenario.** You have been offered the option to receive a free vaccine against the coronavirus. When you decide whether or not to get the vaccine, to what extent are the following considerations likely to be relevant to your thinking?

**Intentions to Vaccinate their Child Scenario.** Your child is in a regular health check-up and the physician asks if you want your kid to get the new vaccine against the coronavirus. When you decide whether or not to let your child(ren) get the vaccine, to what extent are the following considerations likely to be relevant to your thinking?

Table B. Moral Foundations Questionnaire Items in Each Scenario

| Foundation | Item |
| --- | --- |
| **Mask wearing scenario** | |
| Harm to others | Whether someone (apart from myself) is likely to get severely sick. |
| Harm to self | Whether I am likely to get sick. |
| Authority | Whether I am respecting the rules established by my government. |
| Ingroup | Whether I am betraying the values of my community or group of friends. |
| Purity | Whether other people going to the party are likely to be pure and decent. |
| Liberty | Whether I feel I am exercising my liberty. |
| Attention check | Whether someone at the party is likely to be good at math. |
| **Social distancing scenario** | |
| Harm to others | Whether other people’s health is likely to be affected. |
| Harm to self | Whether my health is likely to be affected. |
| Authority | Whether I am respecting the regulations issued by my government. |
| Ingroup | Whether I am staying loyal to the values of my community or group of friends. |
| Purity | Whether I will be violating my own standards of decency. |
| Liberty | Whether I am exercising my personal freedom. |
| Attention check | Whether my friend is wearing a belt. |
| **Intentions to personally vaccinate scenario** | |
| Harm to others | Whether I am reducing the risk of someone (apart from myself) getting severely sick. |
| Harm to self | Whether I am reducing the risk of myself getting sick. |
| Authority | Whether I am respecting the conventions put in place by authorities. |
| Ingroup | Whether my choice demonstrates loyalty to my friends and neighbors. |
| Purity | Whether I will be violating the purity and naturalness of my body. |
| Liberty | Whether I feel I am exercising free will. |
| Attention check | Whether the vaccine comes in a colorful package. |
| **Intentions to vaccinate their child scenario** | |
| Harm to others | Whether the vaccination will protect other children. |
| Harm to self | Whether I will be protecting my child(ren). |
| Authority | Whether I am respecting the norm or expectation established by my government. |
| Ingroup | Whether I would feel disloyal to my community or group’s values. |
| Purity | Whether I will be violating the purity and naturalness of my child(ren). |
| Liberty | Whether I feel I am exercising my personal freedom of choice. |
| Attention check | Whether my child is good at drawing. |

Materials Study 2

Table C. New Items Added to the COVID-19 Engagement in Preventative Effective Behaviors Questionnaire (Study 2)

| Effective behavior | Item |
| --- | --- |
| Wearing a mask | When outdoors around strangers and unable to stay six feet away |
|  | When socializing indoors with an unvaccinated friend who has recovered from COVID-19 (When socializing indoors with a friend who has recovered from COVID-19) |
| Social distancing (reverse coded) | Attend large events (20+ people) outdoors |
|  | Hug other people |
| Washing | Require my child to wash their hands after being outside |

*Note.* Items not listed were carried over from Study 1. Wording adjustments made to some items when asking about the peak in Study 2 are shown in parentheses.

Table D. Modified Items of the COVID-19 Engagement in Preventative Effective and Ineffective Behaviors Questionnaire for Study 2

| Item in Study 1 | Modification for Study 2 |
| --- | --- |
| Effective preventative behaviors | |
| When in a car with people I don’t live with | When in a car with unvaccinated friends ^a^ |
| Attend events with 10 or more people indoors | Attend large events (20+ people) indoors |
| Ineffective preventative behaviors | |
| When outdoors around *other people* but able to stay six feet away | When outdoors around *strangers* but able to stay six feet away |

^a^ Item modified only in the questionnaire about the post-peak (the Study 2 item about the peak remained the same as in Study 1).

Table E. Items in Moderation Analysis from Study 1 (Peak in December) and Study 2 (Post-peak in May)

| Type of behavior | Item |
| --- | --- |
| **Effective preventative behaviors** | |
| Wearing a mask | When grocery shopping |
|  | When taking public transportation |
|  | When in a car with people I don’t live with (When in a car with unvaccinated friends) |
|  | When in school |
| Social distancing (reverse coded) | Shake other people’s hands |
|  | Go to indoor restaurants |
|  | Go to indoor bars |
|  | Attend events with 10 or more people indoors (Attend large events [20+ people] indoors) |
| Washing | Avoid touching my face |
|  | Wash my hands for 20 seconds |
| **Ineffective preventative behaviors** | |
| Wearing a mask | When I am outdoors alone |
|  | When outdoors around other people but able to stay six feet away (When outdoors around strangers but able to stay six feet away) |
|  | When socializing with people I live with |
| Social distancing (reverse coded) | Borrow tools/objects from my neighbor |
|  | Feed my friend's pet when they are not there |
|  | Exercise outdoors when I am unlikely to encounter other people |
| Washing | Use special “antibacterial” soap |
|  | Wash my children’s clothes |
|  | Have my child bathe/shower |
|  | Purify the air in my house |

*Note.* Adjustments made to some items in the composite for Study 2 are shown in parentheses. Items without parentheses were the same in Study 1 and Study 2.

Table F. Score Assigned to Choices about Intention to Vaccinate the Self

| Score for intentions to vaccinate | Choices |
| --- | --- |
| Yes | 1. Yes, I have been fully vaccinated. 2. Yes, I have received my first dose and I am waiting to get my second dose 3. No, but I intend to get vaccinated as soon as I can |
| No | 1. No, and I will only get vaccinated if I can choose which vaccine to get 2. No. I want to wait and see if the vaccines have unforeseen risks 3. No. I will only get vaccinated if it is required (e.g., by my workplace, by the state I live in) 4. No, and I do not intend to get vaccinated even if it’s required |

*Note.* Participants were asked “Now that vaccines are becoming publicly available, are you intending to take the vaccine?”.

Table G. Score Assigned to Choices about Intention to Vaccinate their Child

| Intention to vaccinate | Choices |
| --- | --- |
| Yes | 1. Yes, I want to get my child vaccinated as soon as I can |
| No | 1. No, I want to wait and see if the vaccines have unforeseen risks for children 2. No, I think it’s best to delay vaccines for children until they are older and their immune system is more mature 3. No, I will only get my child vaccinated if it is required (e.g., by the school) 4. No, I do not intend to get my child vaccinated even if it’s required |

*Note.* Participants were asked “When COVID-19 vaccines for children are approved and available, are you intending to have your elementary school-aged child, or children, take the vaccine?”

Supplementary Results

Table H. Mean Moral Foundation Score by Scenario and Country

|  | Mask wearing | | Social distancing | | Vaccination for themselves | | Vaccination for children | |
| --- | --- | --- | --- | --- | --- | --- | --- | --- |
| Foundations | M | SD | M | SD | M | SD | M | **SD** |
| Canada (N=176) | |  |  |  |  |  |  |  |
| Harm to others | 5.18 | 1.28 | 5.3 | 1.18 | 5.26 | 1.25 | 5.12 | 1.31 |
| Harm to self | 4.86 | 1.48 | 5.19 | 1.32 | 5.34 | 1.16 | 5.53 | .97 |
| Authority | 4.86 | 1.39 | 4.72 | 1.49 | 3.86 | 1.72 | 3.74 | 1.73 |
| Ingroup | 3.33 | 1.9 | 3.39 | 1.83 | 2.52 | 1.7 | 2.45 | 1.66 |
| Purity | 2.05 | 1.52 | 3.51 _a_ | 1.98 | 1.94 | 1.5 | 2.02 | 1.61 |
| Liberty | 2.26 | 1.56 | 2.24 | 1.54 | 2.88 | 1.84 | 2.65 | 1.75 |
| United Kingdom (N=139) | | |  |  |  |  |  |  |
| Harm to others | 5.03 | 1.21 | 5.24 | 1.01 | 5.17 | 1.24 | 4.91 | 1.35 |
| Harm to self | 4.82 | 1.32 | 5.17 | 1.15 | 5.09 | 1.24 | 5.49 | .91 |
| Authority | 4.43 | 1.24 | 4.3 | 1.45 | 3.43 | 1.53 | 2.97 | 1.51 |
| Ingroup | 3.39 | 1.71 | 3.21 | 1.64 | 2.35 | 1.53 | 2 | 1.25 |
| Purity | 2.01 | 1.34 | 3.42 _a_ | 1.77 | 1.96 | 1.42 | 2.24 | 1.58 |
| Liberty | 2.37 | 1.36 | 2.48 | 1.51 | 2.99 | 1.63 | 2.8 | 1.62 |
| United States Study 1 (N=138) | | |  |  |  |  |  |  |
| Harm to others | 5.1 | 1.45 | 5.18 | 1.27 | 4.86 | 1.61 | 4.91 | 1.58 |
| Harm to self | 5.12 | 1.44 | 5.23 | 1.29 | 5.16 | 1.34 | 5.64 | .88 |
| Authority | 3.51 | 1.71 | 3.41 | 1.79 | 2.51 | 1.56 | 2.44 | 1.54 |
| Ingroup | 2.99 | 1.81 | 2.96 | 1.8 | 2.12 | 1.47 | 1.78 | 1.2 |
| Purity | 1.96 | 1.4 | 3.51_a_ | 1.95 | 1.99 | 1.54 | 2.38 | 1.78 |
| Liberty | 2.17 | 1.66 | 2.36 | 1.78 | 2.86 | 1.89 | 2.57 | 1.88 |
| United States Study 2 (N=170) | | | | |  |  |  |  |
| Harm to others | 4.94 | 1.45 | 5.04 | 1.42 | 4.93 | 1.55 | 4.68 | 1.68 |
| Harm to self | 4.99 | 1.43 | 5.05 | 1.38 | 5.18 | 1.32 | 5.54 | 0.97 |
| Authority | 3.21 | 1.62 | 3.09 | 1.62 | 2.7 | 1.62 | 2.32 | 1.41 |
| Ingroup | 2.98 | 1.79 | 3.05 | 1.72 | 2.39 | 1.58 | 1.87 | 1.24 |
| Purity | 1.75 | 1.25 | 3.38 _a_ | 1.84 | 1.99 | 1.5 | 2.16 | 1.67 |
| Liberty | 1.96 | 1.38 | 2.25 | 1.59 | 2.72 | 1.75 | 2.35 | 1.7 |

*Note.* The Moral Foundation scores ranged from 1 to 6.

^a^ The purity item in the social distancing situation was removed to improve Cronbach’s alpha.

| Table I. Average Effective Preventative Behaviors in Study 1 and Study 2 (raw scores per item) | | | | | | | | |
| --- | --- | --- | --- | --- | --- | --- | --- | --- |
|  | Canada | | United Kingdom | | United States  Study 1 | | United States  Study 2 | |
| Effective behaviors | M | SD | M | SD | M | SD | M | SD |
| **Mask Wearing** |  |  |  |  |  |  |  |  |
| When grocery shopping | 0.98 | 0.13 | 0.97 | 0.17 | 0.9 | 0.3 | 0.8 | 0.4 |
| When taking public transportation | 0.98 | 0.15 | 0.97 | 0.17 | 0.92 | 0.27 | 0.84 | 0.37 |
| When in a car with people I don’t live with/*When in a car with unvaccinated friends | 0.9 | 0.3 | 0.81 | 0.39 | 0.8 | 0.4 | 0.64 | 0.48 |
| When in school | 0.95 | 0.21 | 0.82 | 0.39 | 0.9 | 0.3 | 0.81 | 0.39 |
| When outdoors around strangers and unable to stay six feet away | / | / | / | / | / | / | 0.64 | 0.48 |
| When socializing indoors with an unvaccinated friend who has recovered from COVID-19 | / | / | / | / | / | / | 0.62 | 0.49 |
| **Social distancing** |  |  |  |  |  |  |  |  |
| Shake other people’s hands | 4.78 | 0.65 | 4.71 | 0.62 | 4.67 | 0.68 | 4.35 | 0.82 |
| Go to indoor restaurants | 4.66 | 0.73 | 4.58 | 0.68 | 4.67 | 0.64 | 4.04 | 1.03 |
| Go to indoor bars | 4.57 | 0.81 | 4.62 | 0.69 | 4.54 | 0.82 | 4.1 | 0.99 |
| Attend events with 10 or more people indoors | 4.69 | 0.76 | 4.74 | 0.62 | 4.69 | 0.68 | 4.22 | 0.96 |
| Hug other people | / | / | / | / | / | / | 4.14 | 0.89 |
| Attend large events (20+ people) outdoors | / | / | / | / | / | / | 4.09 | 1.04 |
| **Washing** |  |  |  |  |  |  |  |  |
| Avoid touching my face | 3.82 | 1.01 | 3.61 | 0.84 | 3.85 | 0.94 | 3.59 | 0.97 |
| Wash my hands for 20 seconds | 4.14 | 0.82 | 4.22 | 0.76 | 4.12 | 0.85 | 3.67 | 0.89 |
| Require my child to wash their hands after being outside | / | / | / | / | / | / | 3.69 | 0.89 |
| Composite of effective behaviors | 0 | 0.63 | 0 | 0.55 | 0 | 0.71 | 0 | 0.71 |

*Note:* The items were standardized, and an average was calculated for the composite.

Table J. Average Ineffective Preventative Behaviors in Study 1 and Study 2 (Raw Scores per Item)

|  | Canada | | United Kingdom | | United States  Study 1 | | United States  Study 2 | |
| --- | --- | --- | --- | --- | --- | --- | --- | --- |
| Ineffective behaviors | M | SD | M | SD | M | SD | M | SD |
| **Mask Wearing** |  |  |  |  |  |  |  |  |
| When I am outdoors alone | 0.15 | 0.36 | 0.09 | 0.29 | 0.12 | 0.33 | 0.06 | 0.24 |
| When outdoors around other people but able to stay six feet away | 0.4 | 0.49 | 0.24 | 0.43 | 0.59 | 0.49 | 0.33 | 0.47 |
| When socializing with people I live with | 0.15 | 0.36 | 0.05 | 0.22 | 0.11 | 0.31 | 0.06 | 0.25 |
| **Social distancing** |  |  |  |  |  |  |  |  |
| Borrow tools/objects from my neighbor | 3.92 | 0.9 | 3.73 | 0.94 | 3.87 | 0.92 | 3.48 | 0.89 |
| Feed my friend's pet when they are not there | 3.68 | 0.93 | 3.64 | 0.91 | 3.65 | 0.9 | 3.35 | 0.8 |
| Exercise outdoors when I am unlikely to encounter other people | 2.89 | 0.98 | 2.88 | 0.9 | 3.12 | 1.08 | 2.79 | 0.92 |
| **Washing** |  |  |  |  |  |  |  |  |
| Use special “antibacterial” soap | 3.59 | 0.89 | 3.77 | 0.84 | 3.7 | 0.85 | 3.37 | 0.8 |
| Wash my children’s clothes | 3.47 | 0.81 | 3.5 | 0.76 | 3.5 | 0.78 | 3.25 | 0.65 |
| Have my child bathe/shower | 3.38 | 0.76 | 3.32 | 0.65 | 3.5 | 0.79 | 3.21 | 0.63 |
| Purify the air in my house | 3.28 | 0.77 | 3.14 | 0.44 | 3.3 | 0.71 | 3.1 | 0.77 |
| Composite of ineffective behaviors | 0 | 0.56 | 0 | 0.54 | 0 | 0.61 | 0 | 0.56 |

*Note:* The items were standardized, and an average was calculated for the composite.

Stability of the Effect of the Moral Foundations across Time Points

As preregistered, we were interested in testing whether the time point of data collection (December peak vs. May pandemic post-peak) moderated the effect of any of the six moral foundations on effective and ineffective preventative behaviors of participants in the US. Therefore, we conducted regression analyses on each of the dependent variables in Study 1 and Study 2 (participants’ effective and ineffective preventative behaviors and vaccine intentions) with the moral foundations as predictors and time point as a moderator variable. For these analyses, we calculated new composites using only the items that were in both Study 1 and Study 2 (see Table E above). The analysis did not reveal any significant moderation effect of time of data collection, suggesting high stability in the patterns of results across time.

Predictors of Change in Preventative Behaviors Between Peak and Post-peak

Additionally, we were interested in comparing people’s reports of their current post-peak behavior and retrospective reports of their behavior at the peak of the pandemic. Therefore, within the second US sample (Study 2 at post-peak), we not only asked participants about their engagement in ineffective and effective behaviors at the current post-peak (“in the US today”), but—using the same items—about their behaviors during the peak (“at the recent peak of the pandemic in the US”). For these analyses, we computed a mean change score across paired items. After standardizing the resulting score for each pair of items, we then created a composite “change in effective behavior” and “change in ineffective behavior” score. Two quantile regressions exploring whether any of the moral foundations predicted change in participants’ effective and ineffective behaviors were non-significant. In fact, there was low variability in self-rated behavioral change between the pandemic peak vs. post-peak.

Is the Effect of Political Ideology on Preventative Behaviors Mediated by Moral Foundations?

Given prior research suggesting a relationship between political ideology and compliance with preventative behaviors [1–3], we decided to more deeply explore the relationship with political ideology within our own data, even though we found no effect when controlling for the six moral foundations. Specifically, we tested whether political ideology had an influence on preventative behaviors through the moral foundations. We conducted four mediation analysis in each country (see Figure A).

***Figure A. Mediation Model with Effective Behaviors as Outcome***


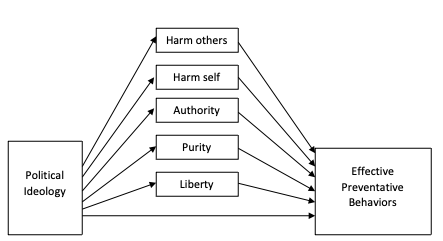


For each of the outcomes (effective behaviors, ineffective behaviors, intentions to vaccinate the self, and intentions to vaccinate the child), we built a model with political ideology as the predictor and each of the six moral foundations as mediators. Bootstrap confidence intervals were calculated to assess the significance of the indirect effects based on 5.000 bootstrap samples. The only significant indirect effects to occur were in Canada and the US.

In the US at the peak of the pandemic (Study 1), the four mediation models were significant (effective behaviors: F[7,130] = 31.69, *p* < .001*, R^2^ =* .63; ineffective behaviors: F[7,130] = 7.93, *p <* .001*, R^2^ =* .30*;* vaccination intentions to the self: *-2LL* = 111.42, *p < .001, Nagelkerke’s R2 = .*50; and vaccination intentions for the child: *-2LL* = 121.92, *p < .001, Nagelkerke’s R2* = .49). In the case of effective preventative behaviors, bootstrap confidence intervals revealed evidence of an indirect effect of political ideology through the foundations of harm to other (*b* = -0.14, 95% CI [-0.27, -0.06]), and liberty (*b =* -0.06, 95% CI [-0.11, -0.01]). Lower conservatism was associated with greater concern about harm to others (*b* = -0.55, *p* < .001), which in turn led to greater engagement in effective preventative behaviors (*b =* 0.26*, p <* .001). Meanwhile, higher conservatism was associated with greater concerns about liberty (*b =* 0.65*, p* < .001) which in turn led to lower reported engagement in effective preventative behaviors (*b* = -0.09*, p* = .004).

In the case of ineffective behaviors, we found an indirect effect of political ideology through the foundation of purity (*b* = 0.04, 95% CI [0.01, 0.09]): Greater conservatism predicted greater concerns about purity (*b = 0.31, p =* .001) which in turn predicted greater reported engagement in ineffective behaviors (*b =* 0.14*, p =* .002). The foundation of purity also mediated the effect of political ideology on intentions to vaccinate the self (*b* = -0.25, 95% CI [-0.61, -0.08]) and a child (*b* = -0.24, 95% CI [-0.56, -0.08]). Given that greater conservatism predicted greater purity considerations, these then, in turn, predicted lower intentions to vaccinate the self (*b =* -0.79*, p = .001*) and the child (*b =* -0.77*, p <* .001). Finally, we also found an indirect effect of political ideology on intentions to vaccinate a child through the foundation of harm others (*b* = -0.38, 95% CI [-1.06, -0.06]). Specifically, as noted above, lower conservatism predicted greater concern about harm to others which then, in turn, predicted greater intentions to vaccinate a child (*b =* 0.70, *p = .*042).

At the peak of the pandemic in Canada, we found very similar results with three models showing significant mediation effects (effective behaviors: F[7,168] = 9.96, *p < .001, R^2^ = .29*; vaccination intentions to the self: -2LL = 105.79, *p* < .001*, Nagelkerke’s R2 = .*53; and vaccination intentions for the child: -2LL = 121.92, *p < .001, Nagelkerke’s R2 =* .49).

A significant indirect effect of political ideology on effective behaviors was mediated by the foundations of harm to others (*b* = -0.06, 95% CI [-0.14, -0.01]) and liberty (*b =* -0.03, 95% CI [-0.07, -0.002]). Specifically, lower conservatism predicted greater concern about harm to others (*b =* -0.28*, p* < .001) which in turn led to greater effective preventative behaviors (*b* = 0.21*, p =* .004). By contrast, greater conservatism was associated with greater liberty considerations (*b =* 0.27, *p* = .008) which, in turn, resulted in lower effective preventative behaviors (*b* = -0.11, p < .001). Liberty considerations mediated the effect of political ideology on vaccine intentions for the self as well (*b* = -0.13, 95% CI [-0.36, -0.004]), given that liberty led to lower intentions to vaccinate the self (*b* = -0.49, p = .012).

As in the US, the purity foundation mediated the effect of political ideology on intentions to vaccinate the self (*b* = -0.24, 95% CI [-0.55, -0.06]) and a child (*b* = -0.17, 95% CI [-0.38, -0.04]). Specifically, greater conservatism was associated with greater concerns about purity (*b =* 0.24*, p =* .008) which led to lower intentions to vaccinate the self (*b* = -0.99, p < .001) and the child (*b* = -0.69, p < .001).

Finally, at the post-peak of the pandemic in the US (Study 2), we found three significant mediation effects (effective behaviors: F[7,162] = 24.62, *p* < .001*, R^2^ =* .52; vaccination intentions to the self: *-2LL* = 111.86, *p < .001, Nagelkerke’s R2 = .*61; and vaccination intentions for the child: *-2LL* = 157.58, *p < .001, Nagelkerke’s R2* = .49).

Consistent with Study 1, at the post-peak the results showed a significant indirect effect of political ideology on effective behaviors through the foundations of harm to others (*b* = -0.07, 95% CI [-0.12, -0.02]) and harm to the self (*b* = -0.07, 95% CI [-0.11, -0.03]). Specifically, lower conservatism predicted greater concern about harm to others (*b =* -0.42*, p* < .001) and harm to the self (*b =* -0.33*, p* < .001) which in turn led to greater effective preventative behaviors (*b* = 0.16*, p =* .001; *b* = 0.01*, p <* .001, respectively).

Similarly, the foundation of harm to the self mediated the effect of political ideology on intentions to vaccinate the self (*b* = -0.28, 95% CI [-0.76, -0.01]) and vaccinate a child (*b* = -0.26, 95% CI [-0.56, -0.05]). As noted, lower conservatism predicted greater concerns about harm to the self and that association then led to greater intentions to vaccinate the self (*b =* -0.85*, p* = .013) and the child (*b =* -0.78*, p* = .027).

Table K. Demographics of Indian Sample

| N | %Women | Age (years) | Race | Socio-economic Political orientation |
| --- | --- | --- | --- | --- |
| 48 | 48% | M=35, SD=6.84 | American Indian or Alaska Native 2%, East Asian 10%, South Asian 79%, Not listed 8% | M=2.47, SD=0.85 |

*Note.* The Socio-economic Political orientation scale ranged from 1 to 5, with greater scores indicating greater conservatism

Table L. Mean Moral Foundation Score by Scenario in India

|  | Mask wearing | | Social distancing | | Vaccination for themselves | | Vaccination for children | | Average across scenarios | |  |
| --- | --- | --- | --- | --- | --- | --- | --- | --- | --- | --- | --- |
| Foundations | M | SD | M | SD | M | SD | M | SD | M | SD |  |
| India (N=48) |  |  |  |  |  |  |  |  |  |  |  |
| Harm to others | 4.56 | 1.53 | 5.06 | 1.12 | 4.75 | 1.33 | 4.67 | 1.17 | 4.76 | 0.98 |  |
| Harm to self | 4.88 | 1.41 | 5.06 | 1.21 | 4.79 | 1.32 | 5.33 | .97 | 5.02 | 1 |  |
| Authority | 5.06 | 1.17 | 4.81 | 1.44 | 4.56 | 1.38 | 4.6 | 1.32 | 4.76 | 0.98 |  |
| Ingroup | 3.73 | 1.85 | 3.69 | 1.67 | 3.12 | 1.63 | 2.73 | 1.67 | 3.18 ^a^ | 1.26 |  |
| Purity | 2.94 | 1.77 | 3.6 | 1.75 | 2.81 | 1.68 | 2.65 | 1.8 | 3 | 1.23 |  |
| Liberty | 3.15 | 1.82 | 3.29 | 1.71 | 3.27 | 1.63 | 3.27 | 1.69 | 3.24 | 1.35 |  |

*Note.* Moral Foundation scores ranged from 1 to 6.

^a^ Average calculated after excluding one item (mask wearing) from the ingroup foundation to improve Cronbach’s Alpha.

Table M. Regression of Dependent Variables on Moral Foundations in India

|  | Effective behaviors | Ineffective behaviors | Vaccinate the self | Vaccinate their child |
| --- | --- | --- | --- | --- |
| Foundations | B (SE) | B (SE) | OR (95% CI) | OR (95% CI) |
| (Intercept) | -0.64 (0.47) | -1.40 (0.42)** | 0.13 (0.00 – 29.53) | 0.14 (0.00 – 7.84) |
| Harm to others | 0.14 (0.14) | 0.02 (0.13) | 1.81 (0.33 – 9.91) | 2.79 (0.75 – 14.68) |
| Harm to self | 0.05 (0.14) | 0.05 (0.13) | 1.61 (0.37 – 6.98) | 0.56 (0.14 – 2.00) |
| Authority | 0 (0.09) | 0.16 (0.09) | 1.2 (0.36 – 3.91) | 2.02 (0.87 – 5.18) |
| Ingroup | -0.03 (0.07) | 0.13 (0.07) | 3.03 (1.02 – 11.23) | 0.83 (0.39 – 1.72) |
| Purity | 0 (0.07) | 0.12 (0.09) | 0.26 (0.04 – 1.07) | 0.66 (0.26 – 1.49) |
| Liberty | -0.01 (0.07) | -0.15 (0.08)* | 0.78 (0.22 – 2.83) | 0.85 (0.39 – 1.90) |
| Regression | Quantile | Linear | Logistic | Logistic |

*Note.* One item was excluded from the ineffective behavior composite to improve Cronbach’s Alpha reliability

* *p <.*05*, *** *p <.*01, *** *p <.*001

**References**

[1] Gollwitzer A, Martel C, Brady WJ, Pärnamets P, Freedman IG, Knowles ED, et al. Partisan differences in physical distancing are linked to health outcomes during the COVID-19 pandemic. Nat Hum Behav 2020;4:1186–97. https://doi.org/10.1038/s41562-020-00977-7.

[2] Pennycook G, McPhetres J, Bago B, Rand DG. Beliefs About COVID-19 in Canada, the United Kingdom, and the United States: A Novel Test of Political Polarization and Motivated Reasoning. Pers Soc Psychol Bull 2021:014616722110236. https://doi.org/10.1177/01461672211023652.

[3] Stroebe W, vanDellen MR, Abakoumkin G, Lemay EP, Schiavone WM, Agostini M, et al. Politicization of COVID-19 health-protective behaviors in the United States: Longitudinal and cross-national evidence. PLOS ONE 2021;16:e0256740. https://doi.org/10.1371/journal.pone.0256740.
